# Generation of a Spindle Checkpoint Arrest from Synthetic Signaling Assemblies

**DOI:** 10.1016/j.cub.2016.11.014

**Published:** 2017-01-09

**Authors:** Ivan Yuan, Ioanna Leontiou, Priya Amin, Karen M. May, Sadhbh Soper Ní Chafraidh, Eliška Zlámalová, Kevin G. Hardwick

**Affiliations:** 1 Wellcome Trust Centre for Cell Biology, University of Edinburgh King’s Buildings, Max Born Crescent, Edinburgh EH9 3BF, UK

**Keywords:** checkpoint, spindle, mitosis, kinetochore, Mad, Bub, Mps1, synthetic, cell cycle

## Abstract

The spindle checkpoint acts as a mitotic surveillance system, monitoring interactions between kinetochores and spindle microtubules and ensuring high-fidelity chromosome segregation [1, 2, 3]. The checkpoint is activated by unattached kinetochores, and Mps1 kinase phosphorylates KNL1 on conserved MELT motifs to generate a binding site for the Bub3-Bub1 complex [4, 5, 6, 7]. This leads to dynamic kinetochore recruitment of Mad proteins [8, 9], a conformational change in Mad2 [10, 11, 12], and formation of the mitotic checkpoint complex (MCC: Cdc20-Mad3-Mad2 [13, 14, 15]). MCC formation inhibits the anaphase-promoting complex/cyclosome (Cdc20-APC/C), thereby preventing the proteolytic destruction of securin and cyclin and delaying anaphase onset. What happens at kinetochores after Mps1-dependent Bub3-Bub1 recruitment remains mechanistically unclear, and it is not known whether kinetochore proteins other than KNL1 have significant roles to play in checkpoint signaling and MCC generation. Here, we take a reductionist approach, avoiding the complexities of kinetochores, and demonstrate that co-recruitment of KNL1^Spc7^ and Mps1^Mph1^ is sufficient to generate a robust checkpoint signal and prolonged mitotic arrest. We demonstrate that a Mad1-Bub1 complex is formed during synthetic checkpoint signaling. Analysis of *bub3*Δ mutants demonstrates that Bub3 acts to suppress premature checkpoint signaling. This synthetic system will enable detailed, mechanistic dissection of MCC generation and checkpoint silencing. After analyzing several mutants that affect localization of checkpoint complexes, we conclude that spindle checkpoint arrest can be independent of their kinetochore, spindle pole, and nuclear envelope localization.

## Highlights

• Synthetic signaling scaffolds generate a spindle checkpoint arrest
• The combination of KNL1^Spc7^ and Mps1^Mph1^ kinase generates a robust arrest
• Kinetochore, spindle, and nuclear envelope enrichment of the scaffold is not required
• Bub3 acts to inhibit premature checkpoint activation

## Graphical Abstract

**Figure:**
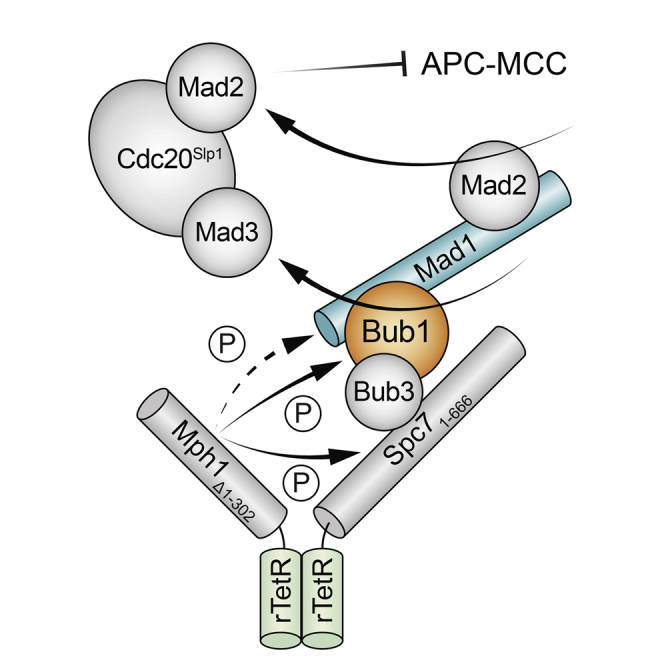

## Teaser

Yuan et al. employ synthetic protein assemblies to generate a robust spindle checkpoint arrest in fission yeast. Formation of artificial heterodimers between a checkpoint kinase and one of its key substrates is shown to be sufficient for mitotic arrest, entirely independently of the location of this complex in the yeast nucleus.

## Results and Discussion

**Figure 1:**
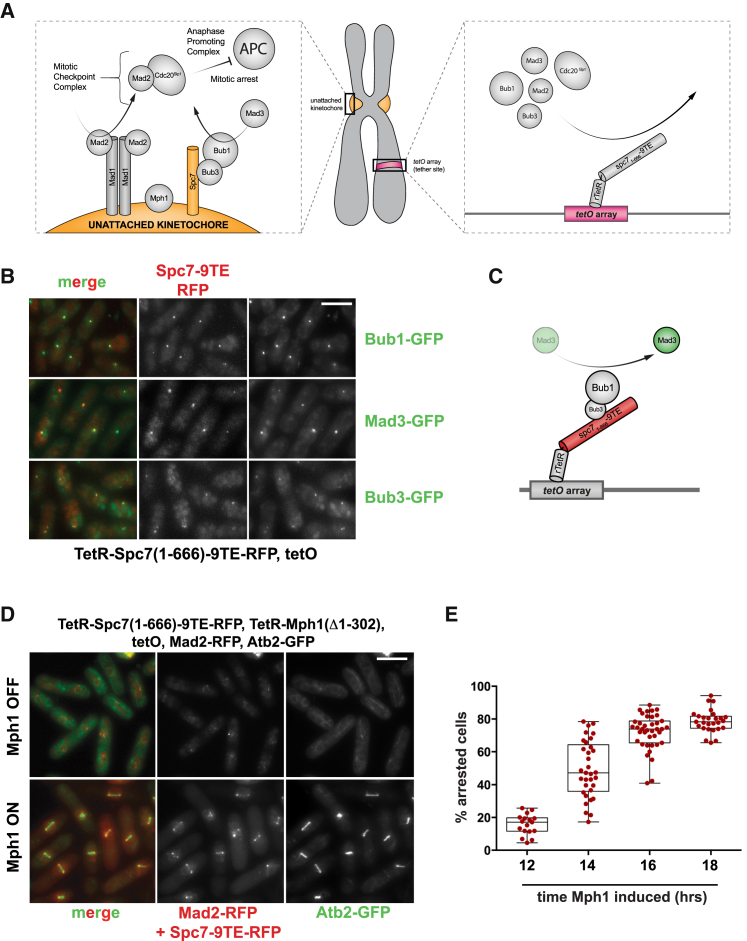
Co-tethering of Spc7-9TE and TetR-Mph1ΔN Generates a Robust Checkpoint Arrest (A) Schematic model of kinetochore-based checkpoint signaling versus the synthetic tetO platform. (B) TetR-Spc7-9TE is sufficient to recruit Bub1-GFP, Bub3-GFP, and Mad3-GFP to an array of Tet operators on a chromosome arm. Scale bar, 10 μm. See Figure S1 for TetR-Spc7wt and TetR-Spc7-9TA images. (C) Schematic summary of Spc7-9TE tethering. (D) Co-expression of TetR-Spc7-9TE and TetR-Mph1ΔN produces a robust mitotic arrest with short metaphase spindles. Scale bar, 10 μm. (E) Quantitation of arrested cells after 12, 14, 16, and 18 hr of Mph1 induction (cells grown without thiamine). The plus thiamine control culture does not arrest, containing just a few mitotic cells. 36 experiments were performed and data points are plotted along with the mean and SD. See also Figure S1.

Genetic and proteomic approaches have revealed that kinetochores are highly complex molecular machines (with ∼100 kinetochore components in vertebrates [16] and ∼50 in yeast [17]) and that there are approximately ten components of the spindle checkpoint machinery [2]. Amidst such complexity, separating kinetochore bi-orientation, error-correction, and microtubule attachment functions from true checkpoint activation and signaling functions is problematic. Kinetochore tethering of, e.g., Mph1-Ndc80 and Mis12-Mad1 can initiate checkpoint arrests [18, 19]. However, it is very likely that in such experiments endogenous kinetochore function is being perturbed and that these perturbations then activate the spindle checkpoint, making interpretation of the experiments complicated and rather unsatisfactory. To improve on this tethering strategy, we set out to generate a spindle checkpoint arrest from a site quite distinct from an unattached kinetochore. We employed a fission yeast strain with 112 tandem repeats of the tet operator (tetO) integrated on the arm of chromosome 1 (at the *arg3* locus, see Figure 1A). This is ∼1.5 Mb away from cen1 and can thus be imaged as a distinct spot in live fission yeast cells (see Figure S1A). When we expressed the phosphomimic mutant Spc7(1-666)-9TE fused to the Tet repressor in these cells it resulted in constitutive recruitment of Bub1, Bub3, and Mad3 to the tetO array, throughout the cell cycle and independently of Mph1 kinase (Figure 1B). Note, this fusion protein only contains the first half of Spc7 (1-666) and so completely lacks its C-terminal kinetochore targeting domain. Expression of TetR-Spc7-9TA failed to recruit checkpoint proteins to the tetO array (see Figure S1B), whereas wild-type TetR-Spc7 was able to recruit Bub1, Bub3, and Mad3 but at much lower levels than TetR-Spc7-9TE and in a way that was dependent on endogenous Mph1 kinase action (see Figure S1B). This demonstrates that the “activated” Spc7-9TE binding platform is sufficient to recruit these three checkpoint proteins constitutively, and that this works ectopically and thus does not require additional kinetochore factors. Bub1p, Bub3p, and Mad3p are recruited to the array with the expected dependencies (see Figures S1C–S1E): thus, we believe that this Spc7-Bub-Mad3 complex likely acts as an independent signaling module (Figure 1C).

### Co-tethering KNL1^Spc7^ and Mps1^Mph1^ Kinase Generates a Robust Mitotic Arrest

**Figure 2:**
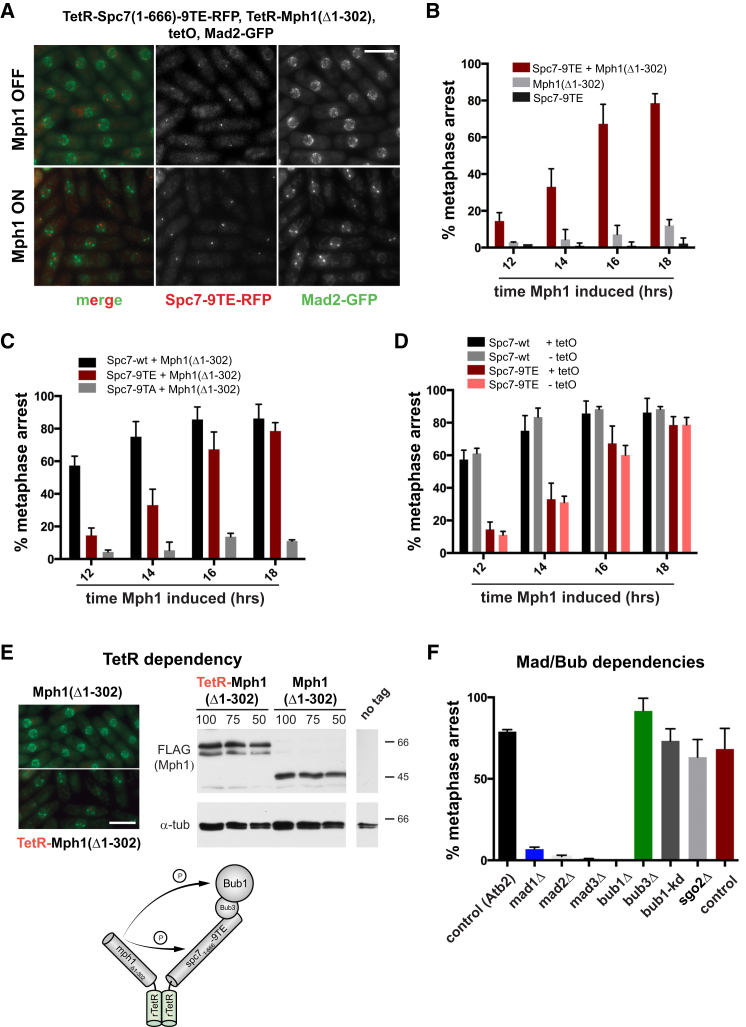
Dependencies for Synthetic Checkpoint Arrest (A) Co-expression of TetR-Spc7-9TE and TetR-Mph1ΔN leads to a metaphase arrest with Mad2-GFP accumulating at the spindle poles (analyzed in detail in Figure 3). Scale bar, 10 μm. (B) Expression of either TetR-Spc7-9TE or TetR-Mph1ΔN alone is not sufficient for robust arrest. This experiment was repeated three times and is plotted as the mean ± SD. (C) Comparison of TetR-Spc7-9TE, TetR-wild-type Spc7 (Spc7-wt), and TetR-Spc7-9TA. The latter is unable to arrest cells, whereas the wild-type protein arrests better than Spc7-9TE. This experiment was repeated three times and is plotted as the mean ± SD. (D) The tetO array is not necessary for Mad2-GFP accumulation at spindle poles or metaphase arrest. The mitotic arrest, for both TetR-Spc7-wt and TetR-Spc7-9TE, was compared in strains containing either 112xtetO or no tet operators. This experiment was repeated three times and is plotted as the mean ± SD. (E) No arrest was observed when TetR was removed from the Mph1 fusion protein (Mad2-GFP did not accumulate at spindle poles). Scale bar, 10 μm. Anti-Flag (Mph1) immunoblot of whole cell extracts demonstrates that similar levels of Mph1 were expressed with and without TetR. (F) The mitotic arrest is Mad1, Mad2, Mad3, and Bub1 dependent, but independent of Bub3, Bub1 kinase activity, and Sgo2. These strains were analyzed at least three times and data plotted as the mean ± SD. The control strain (TetR-Spc7-9TE) on the left has Atb2-GFP as reporter and on the right Mad2-RFP. All strains contained the tetO array, apart from *sgo2*Δ and its corresponding control strain. Representative images are presented in Figure S2J. See also Figure S2.

**Figure 3:**
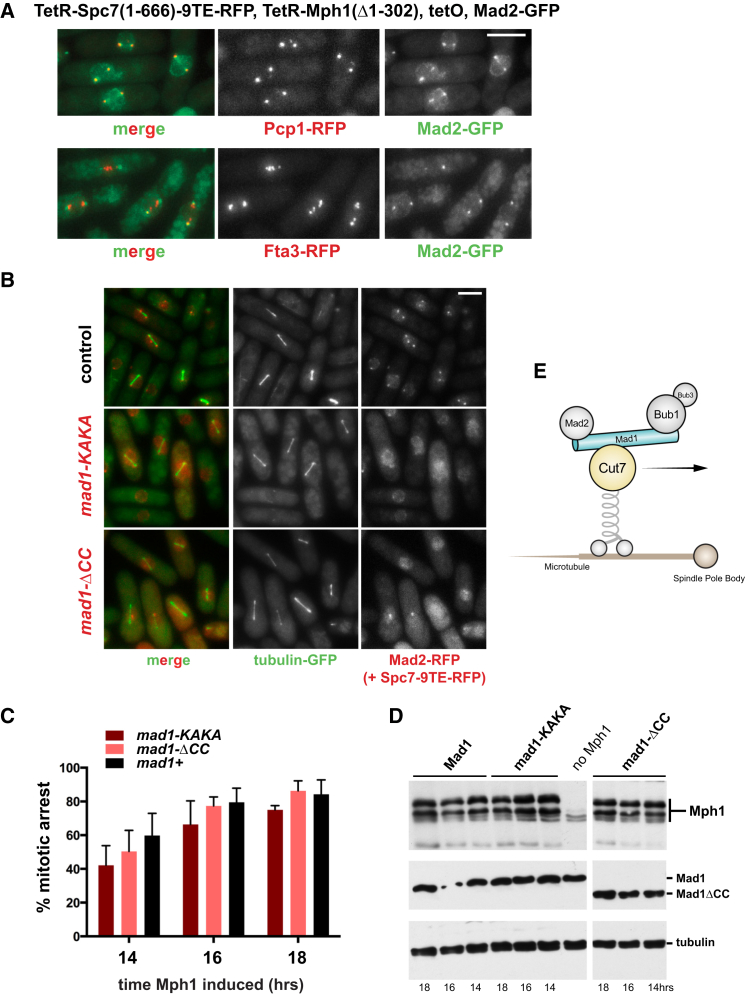
Mad2p Accumulates at Spindle Poles in the Synthetic Arrest, but This Is Not Necessary for the Arrest (A) Cells were arrested with co-expression of TetR-Mph1ΔN and TetR-Spc7-9TE. Co-localization of the spindle pole marker Pcp1-RFP and Mad2-GFP is observed. The Mad2-GFP does not co-localize well with the kinetochore marker Fta3-RFP in arrested cells, although in a few cases kinetochores are close to the poles. Scale bar, 5 μm. Figure S3A demonstrates co-immunoprecipitation of Mad2 with gamma tubulin complex proteins. (B) Strains co-expressing Spc7 and Mph1 do not accumulate Mad2-GFP at spindle poles in strains containing the *mad1-KAKA* mutation that disrupts the Mad1-Cut7 kinesin motor interaction. Other motor mutants were analyzed (*dynein*, *klp2*Δ, *klp5/6*Δ) but found to have no effect on the arrest or Mad2-GFP localization to spindle poles (see Figure S3G). Scale bar, 10 μm. The *mad1-*Δ*CC* allele still arrests even though localization of Mad1 and Mad2 to the nuclear periphery/envelope and spindle poles is lost. This N-terminal coiled-coil domain also includes the Cut7 interaction site. (C) Quantitation of the *mad1-KAKA* and *mad1-*Δ*CC* mutant arrests. This experiment was repeated five times and data plotted as the mean ± SD. (D) The levels of Mph1 expression and Mad1 protein stability are not affected in these *mad1* mutants. Time of Mph1 induction (after thiamine wash-out) is indicated. (E) Model with the Cut7 kinesin moving the Mad-Bub complex to spindle poles. This predicts that the movement of Bub1 to spindle poles is Bub3 independent, which was found to be the case (see Figure S3D). See also Figure S3.

At unattached kinetochores, Bub1 is thought to recruit Mad1 [20]. However, when we expressed TetR-Spc7-9TE no detectable Mad1-Mad2 proteins were recruited to the array, and no cell-cycle delay was observed (data not shown). When we co-expressed TetR-Mad1 with TetR-Spc7-9TE, again no cell-cycle effects were observed (data not shown). This suggests that co-recruitment of Mad1 and Bub1 is not sufficient for checkpoint signaling at least on this tetO platform. We thought that this might be because the synthetic signaling scaffold assembled there (Spc7-Bub-Mad) lacked Mph1 kinase. Therefore, instead of Mad1, we co-expressed TetR-Spc7-9TE with TetR-Δ(1-302)Mph1, being very careful not to overexpress Mph1 kinase. We particularly wanted to avoid activating the checkpoint from kinetochores, and so the N terminus of Mph1 was removed to prevent this kinase from targeting to endogenous kinetochores [21] where it might be activated and could then recruit other checkpoint complexes. Figures 1D and 1E show a very striking result: co-expression of TetR-Spc7-9TE with TetR-Δ(1-302)Mph1 was sufficient to arrest cells in mitosis. This is seen very clearly in Figure 1D where we used GFP-labeled tubulin to image the short metaphase spindles in arrested cells. Figure 1E shows that, after 16 hr of Mph1 induction, we typically see ∼80% metaphase cells (cf. ∼5% in strains not inducing Mph1, +thiamine). When we imaged Mad2-GFP/RFP in the arrested cells, we saw that, rather than accumulate at the tetO array with the Bub proteins, Mad2-GFP accumulated at the poles of the metaphase spindles (Figure 2A, and see Figure 3A for co-localization). Importantly, this arrest requires co-expression of both TetR-Spc7-9TE and TetR-Δ(1-302)Mph1: neither alone is sufficient for an arrest (Figures 2B and S2A–S2D), and their arrest does not require endogenous Mph1 kinase (Figures S2D–S2G).

Next, we analyzed Spc7-wt and Spc7-9TA: while Spc7-9TA had little effect on the cell cycle, we were surprised to find that Spc7-wt arrested significantly faster than Spc7-9TE (Figure 2C), with ∼60% mitotic arrest after 12 hr compared to 16 hr for Spc7-9TE. To analyze this in more detail, we compared Spc7-wt and Spct-9TE arrests in strains expressing TetR-Δ(1-302)Mph1 both with and without endogenous Mph1 kinase. Figure S2D confirms that the wild-type form of this signaling scaffold is more efficient than the Spc7-9TE phosphomimic at generating a checkpoint signal. There are several possible reasons for this: perhaps the nine glutamic acid residues do not fully mimic phosphorylation, or perhaps having all nine sites modified on a single molecule is not optimal for scaffolding function (see Mad2 recruitment below).

Next, we wanted to test what level of co-enrichment of TetR-Spc7-9TE and TetR-Δ(1-302)Mph1 was necessary for initiation of an arrest (each yeast kinetochore is thought to have approximately five molecules of Spc7 [22]), and so we modified our strains by reducing the number of tet operators present, and thus the number of Spc7 and Mph1 binding sites. Strains containing four tandem copies of tetO arrested well (data not shown) and to our surprise so did strains without any tetO sequences at all (Figures 2D and S2J). Consistent with this observation, we found that addition of anhydro-tetracycline (aTc), which enhances TetR binding to the tetO array in this system, had no significant effect on this arrest (Figure S2H). Our interpretation is that soluble, heterodimeric complexes formed between TetR-Spc7 and TetR-Mph1 in the nucleoplasm are sufficient for checkpoint activation. To test this directly, we removed TetR from the Mph1 construct: the resulting strains no longer arrest, and Mad2-GFP does not accumulate at spindle poles (Figure 2E). We conclude that forced interaction of these two critical upstream checkpoint components is sufficient for activation of the spindle checkpoint, and that their enrichment at the tetO array is unnecessary for these signals to induce a metaphase arrest.

If these arrests reflect a normal mode of checkpoint signaling, they should be dependent on downstream checkpoint components. We tested the dependence of this metaphase arrest on the Mad/Bub proteins and found that it required Mad1, Mad2, Mad3, and Bub1. Importantly it did not require “upstream” kinetochore-based signaling: the arrest was independent of endogenous Mph1 and Bub1 kinase activities, of Sgo2, and of Bub3 (Figures 2F and S2I–S2J). The latter is not surprising, as Bub3 is known to be unnecessary for fission yeast spindle checkpoint arrests [23, 24].

### Arrested Cells Accumulate Several Checkpoint Proteins at Their Spindle Poles

Co-expression of Mps1 kinase and an Spc105 fragment has previously been demonstrated to induce a cell-cycle delay in budding yeast [25]. In that study, the rapamycin-induced heterodimers (of Mps1-Spc105) usually became enriched at endogenous kinetochores, which could then serve as a platform to generate or amplify the checkpoint signal. Some evidence was presented suggesting that the cell-cycle delay could be generated independently of kinetochores, using the *ndc10-1* mutation where kinetochores are thought to be destroyed at the restrictive temperature. The possible role of endogenous kinetochores is an important issue, and one we were keen to avoid in our system: our Mph1 construct lacks the N-terminal 302 residue kinetochore targeting domain, our Spc7 construct lacks the C-terminal half of the protein that targets it to kinetochores, and most of our strains lack endogenous Mph1 kinase, thereby preventing all the Mad/Bub proteins from being recruited to endogenous kinetochores [21]. We carried out co-localization experiments with kinetochore (Fta3 [26]) and spindle pole (Pcp1 [27]) markers in our arrested fission yeast cells. Figure 3A demonstrates that Mad2-GFP was not recruited to endogenous fission yeast kinetochores but instead overlapped well with gamma-tubulin and spindle pole body markers. Mad1 and Mad2 proteins have been observed at spindle poles previously, and direct interaction with the gamma-tubulin protein Alp4 and Mad2 has been described in fission yeast cells late in mitosis (post-metaphase), but its roles there remain unclear [28]. Co-immunoprecipitation confirms that Mad2-GFP interacts with Alp4 in these synthetically arrested cells (see Figure S3A). We analyzed which other checkpoint proteins were enriched at spindle poles in the arrested cells, by crossing in GFP-tagged forms of Mad1, Mad3, Bub3, and Bub1 (Figures S3B–S3D). Mad3-GFP and Bub1-GFP were recruited both to the tetO array and to spindle poles in cells co-expressing TetR-Spc7-9TE with TetR-Δ(1-310)Mph1 (see Figures S3B–S3D), as is Bub3-GFP (data not shown). Interestingly, Bub1-GFP recruitment to spindle poles did not require Bub3. Similar observations were made with TetR-Spc7-wt experiments with one important exception: in cells co-expressing TetR-Spc7-wt with TetR-Δ(1-310)Mph1, we could also detect Mad2-GFP on the tetO array (Figure S2E). This interesting observation might explain why these cells arrest faster than Spc7-9TE, as it suggests that the Mad1-Mad2 complex associates more stably with the TetR-Spc7-wt platform than with TetR-Spc7-9TE and that this stable complex may then be better able to generate the mitotic checkpoint complex (MCC) and inhibit the anaphase-promoting complex/cyclosome (APC/C).

### Spindle Pole Localization Is Not Necessary for Checkpoint Arrest

We wanted to test whether the spindle pole localization was relevant to generation of the checkpoint arrest in these cells. In human cells, checkpoint proteins are stripped from the outer kinetochore upon microtubule attachment and transported to spindle poles in a dynein-dependent fashion [29]. This is thought to be one way vertebrate cells silence the spindle checkpoint, although it is not essential for silencing [30]. However, there is no evidence that dynein is involved in checkpoint protein targeting in yeast mitosis [31]. We tested dynein, *klp2*, *klp5*, and *klp6* mutants and found no effect on Mad2 localization in our synthetic checkpoint strain (see Figure S3G). An interaction between Mad1 and Cut7 (fission yeast Kinesin 5) was recently reported by Watanabe et al. [32]. They found that recruitment of Cut7 to kinetochores was Mad1 dependent, and that this interaction could be disrupted through mutation of the Mad1 N terminus (with the *mad1-KAKA* mutation) without affecting spindle checkpoint function. We note that the Cut7 kinesin motor has been demonstrated to be bi-directional in vitro [33] and that this motor localizes to spindle poles in addition to the spindle and midzone [34]. When we introduced the *mad1-KAKA* allele into our synthetic checkpoint system, we observed a dramatic decrease in spindle pole localization of Mad2-GFP (Figure 3B). Our interpretation is that fission yeast kinesin 5 is required for spindle pole enrichment of spindle checkpoint proteins in the synthetic arrest. However, imaging revealed that the *mad1-KAKA* cells still efficiently arrested at metaphase, with a diffuse nuclear pool of Mad2-RFP (Figures 3B and 3C). Thus, spindle pole enrichment of checkpoint proteins is not critical for the synthetic arrest, and we conclude that spindle poles are unlikely to be an important site of MCC generation in these cells. Mad1 and Mad2 interact with the nuclear periphery, via Mlp/TPR protein interactions [35, 36], and this has been demonstrated to be an important site of MCC assembly early in vertebrate mitosis [37]. Therefore, we analyzed another *mad1* mutant where the first 136 amino acids of Mad1 containing a coiled-coil region (CC) were removed, preventing Mad1-Mad2 interaction with Mlps and the nuclear envelope and also removing the Cut7 interaction site. These *mad1*-ΔCC cells were also able to arrest efficiently when TetR-Spc7-9TE and TetR-Δ(1-302)Mph1 were co-expressed (Figure 3E). We conclude that the Mad and Bub proteins do not need to be enriched at kinetochores, spindle poles, or the nuclear periphery for a robust checkpoint arrest to be generated in fission yeast. Most likely a diffuse, soluble pool of Spc7-Bub-Mad signaling assemblies is sufficient.

### Checkpoint Signaling Generates a Mad1-Bub1 Complex and Is Inhibited by Bub3

**Figure 4:**
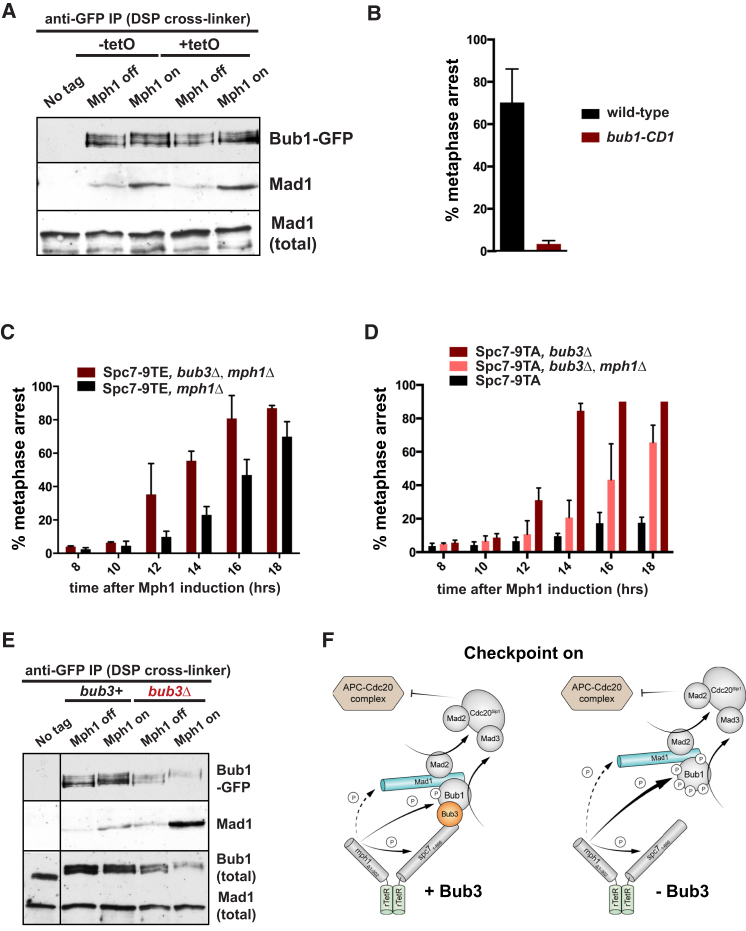
Synthetic Checkpoint Arrest Requires Mad1-Bub1 Complex Formation and Is Inhibited by Bub3 (A) Cells containing Bub1-GFP, TetR-Spc7-9TE, and TetR-Mph1ΔN were arrested as above for 16 hr then cross-linked with DSP before harvesting. Mad1 co-immunoprecipitates with Bub1-GFP in the arrested cells, both in the presence and absence of the tetO array. On the immunoblots, Bub1-GFP was detected with anti-Bub1 antibody and Mad1 with anti-Mad1 (see Supplemental Experimental Procedures). (B) There is no arrest in the *bub1-CD1* mutant, which disrupts the Bub1-Mad1 interaction (see Figure S4A for images). This experiment was repeated three times, and data were plotted as the mean ± SD. The mutant Bub1-CD1 protein is stable (see Figure S4B). (C) *bub3*Δ mutants containing TetR-Spc7-9TE arrest significantly faster than *bub3*^*+*^, TetR-Spc7-9TE. This experiment was repeated three times, and data were plotted as the mean ± SD. (D) TetR-Spc7-T9A combined with TetR-Mph1ΔN is able to arrest cells in the absence of Bub3. This experiment was repeated three times, and data were plotted as the mean ± SD. (E) Higher levels of the Mad1-Bub1 complex are generated in *bub3*Δ cells. These cells contained TetR-Mph1ΔN and TetR-Spc7-9TE and were harvested after 12 hr of TetR-Mph1 induction. On the immunoblots, Bub1-GFP was detected with polyclonal anti-Bub1 antibodies, and Mad1 with anti-Mad1 antibodies (see Supplemental Experimental Procedures). (F) Working model: diffusible heterodimers of TetR-Mph1ΔN and TetR-Spc7(1-666) actively produce a phospho-dependent Bub1-Mad1 complex, that than acts as an assembly platform for MCC production. In the absence of Bub3, shown to the right, Bub1 becomes hyperphosphorylated, which can enhance Bub1-Mad1 complex assembly and MCC production. See also Figure S4.

A biochemical hallmark of active spindle checkpoint signaling in budding yeast is formation of a Bub1-Mad1 complex [20, 38], but this complex has proved challenging to detect in other systems. We immunoprecipitated Bub1-GFP from synthetically arrested cells (both with and without a tetO array), after cross-linking with dithiobis[succinimidylpropionate] (DSP) and were able to pull down complexes containing Mad1 and Mad2 (Figure 4A; data not shown). While our previous experiments suggested that this complex is rather labile in fission yeast extracts, we have also been able to co-immunoprecipitate these proteins in extracts made from *nda3* arrested cells after DSP cross-linking (data not shown). We propose that the synthetic checkpoint arrest is generated from a TetR-Spc7-Bub1 platform and that co-tethered TetR-Mph1 kinase then activates this further by phosphorylating Bub1 [20] to recruit the Mad1-Mad2 complex (Figure 4F). To directly test the importance of the Bub1-Mad1 interaction, we used the *bub1-CD1* mutant, where conserved phospho-sites thought to be needed for Mad1 interaction have been mutated to alanine [20, 39], and we found that these cells were unable to checkpoint arrest (Figure 4B). Co-immunoprecipitation experiments confirm that the Mad1-Bub1 interaction is efficiently generated from the TetR-Spc7-wt platform (data not shown), consistent with our ability to detect Mad2-GFP on the tetO array in the cells with tethered TetR-Spc7-wt (see Figure S2E). Detailed structural studies will be needed to explain this intriguing, partial “separation of function” with the Spc7-9TE allele: it recruits Bub1 better than Spc7-wt to the tetO array (Figure 1), yet it is less effective at recruiting Mad1&2 than Spc7-wt.

Watanabe et al. proposed that Bub3 might act as a chaperone to “suppress the ectopic activation of non-kinetochore Bub1” [6]. If so, one would expect to see a significant effect on the efficiency of ectopic TetR-Spc7-TetR-Mph1-induced checkpoint arrest in *bub3*Δ cells. Consistent with this prediction, Figure 4C demonstrates a striking advance (by ∼4 hr) in the timing of arrest in *bub3*Δ cells arresting due to Spc7-9TE cells (although there is no effect with Spc7-wt, see Figure S4C). Deletion of *bub3* even allowed TetR-Spc7-9TA, TetR-Δ(1-302)Mph1 to arrest cells, again demonstrating the inhibitory effect of Bub3 (Figure 4D). Figure 4E shows a corresponding increase in the level of the Mad1-Bub1 complex in *bub3*Δ cells. We also note that in *bub3*Δ cells Bub1-GFP becomes hyperphosphorylated during mitotic arrest. This suggests one possible mode of Bub3 action: Bub3 binding might inhibit Bub1 auto-phosphorylation and thereby negatively impact Mad1p binding (see model in Figure 4F). We conclude that Bub3 likely acts to prevent ectopic spindle checkpoint signaling. Future experiments will address whether it does this by inhibiting the checkpoint activation pathway, or enhancing spindle checkpoint silencing [24]. In normal cells, Bub3 would prevent early nucleoplasmic signaling, and this effect would later be overcome when Mad-Bub complexes assemble at kinetochores and Spc7-Bub3-Bub1 interactions induce conformational changes in the Bub proteins, thereby activating Bub1 for downstream signaling. These Bub3 findings from our synthetic arrest are entirely consistent with a recent study published while our manuscript was in revision [40].

### Conclusions

We have assembled a simple, synthetic, signaling system (SynCheck), avoiding the complexities of kinetochores, and generated a robust checkpoint arrest in fission yeast cells. KNL1^Spc7^ acts as a platform to recruit Bub complexes and co-targeted Mps1^Mph1^ kinase is sufficient to activate them for downstream signaling. This leads to assembly of a Mad1-Bub1 complex, MCC generation, and metaphase arrest. We note that the resulting cells arrest for several hours and eventually *cut* and die. This is possibly due to inefficient checkpoint silencing and that is currently under investigation.

It is clear from this and previous studies that checkpoint signals can be initiated from several sites: kinetochores, nuclear pores, possibly spindle poles, a tetO array, and soluble hetero-dimers of KNL1^Spc7^-Mps1^Mph1^ in the nucleoplasm. For a field that often equates kinetochore localization with checkpoint action, it is rather humbling to observe that none of this localized enrichment is necessary for checkpoint arrest, at least in the relatively small yeast cells studied here. It will be very interesting to see whether similar ectopic platforms can arrest larger vertebrate cells and, if so, whether apoptosis is induced as this could have therapeutic implications.

## Author Contributions

This project was conceived by K.G.H. Most of the experiments were carried out by I.L. and I.Y., with the exception of *mad1*Δ*CC* and some *bub3*Δ experiments (K.M.M.), Bub1-Mad1 co-immunoprecipitations (P.A.), some *bub3*Δ, *sgo2*Δ and Cen2-GFP experiments (S.S.N.C. and I.L.), and the kinesin and dynein motor mutant analyses (E.Z. and I.L.). The manuscript was written by K.G.H. with help from I.Y., I.L., K.M.M., P.A., and S.S.N.C.

## Supplemental Information

Document S1. Figures S1–S4 and Supplemental Experimental Procedures

Document S2. Article plus Supplemental Information
