# Supplementary material for: Generation of a Spindle Checkpoint Arrest from Synthetic Signaling Assemblies
Source: Curr Biol. 2017 Jan 9;27(1):137–43. doi: 10.1016/j.cub.2016.11.014 (PMC5226922; doi:10.1016/j.cub.2016.11.014)
Supplement: Document S1. Figures S1–S4 and Supplemental Experimental Procedures [file mmc1.pdf]

Current Biology, Volume *27*

Supplemental Information

Generation of a Spindle Checkpoint Arrest from Synthetic Signaling Assemblies

Ivan Yuan, Ioanna Leontiou, Priya Amin, Karen M. May, Sadhbh Soper Ní Chafraidh, Eliška Zlámalová, and Kevin G. Hardwick

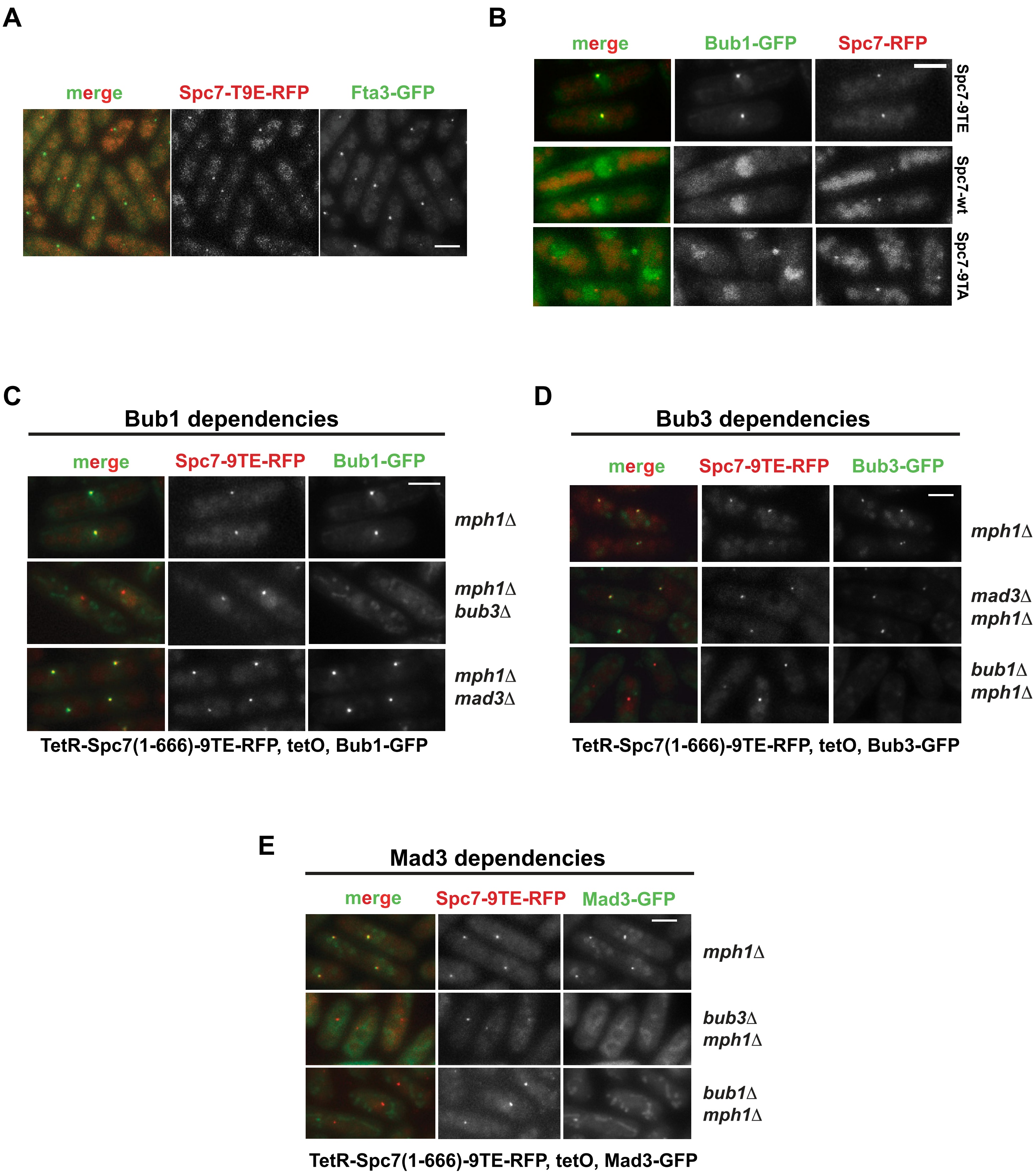
A
merge
Spc7-T9E-RFP
Fta3-GFP
B
merge
Bub1-GFP
Spc7-RFP
Spc7-9TE
Spc7-wt
Spc7-9TA
C
Bub1 dependencies
merge
Spc7-9TE-RFP
Bub1-GFP
mph1Δ
mph1Δ bub3Δ
mph1Δ mad3Δ
TetR-Spc7(1-666)-9TE-RFP, tetO, Bub1-GFP
D
Bub3 dependencies
merge
Spc7-9TE-RFP
Bub3-GFP
mph1Δ
mad3Δ mph1Δ
bub1Δ mph1Δ
TetR-Spc7(1-666)-9TE-RFP, tetO, Bub3-GFP
E
Mad3 dependencies
merge
Spc7-9TE-RFP
Mad3-GFP
mph1Δ
bub3Δ mph1Δ
bub1Δ mph1Δ
TetR-Spc7(1-666)-9TE-RFP, tetO, Mad3-GFP

Figure S1

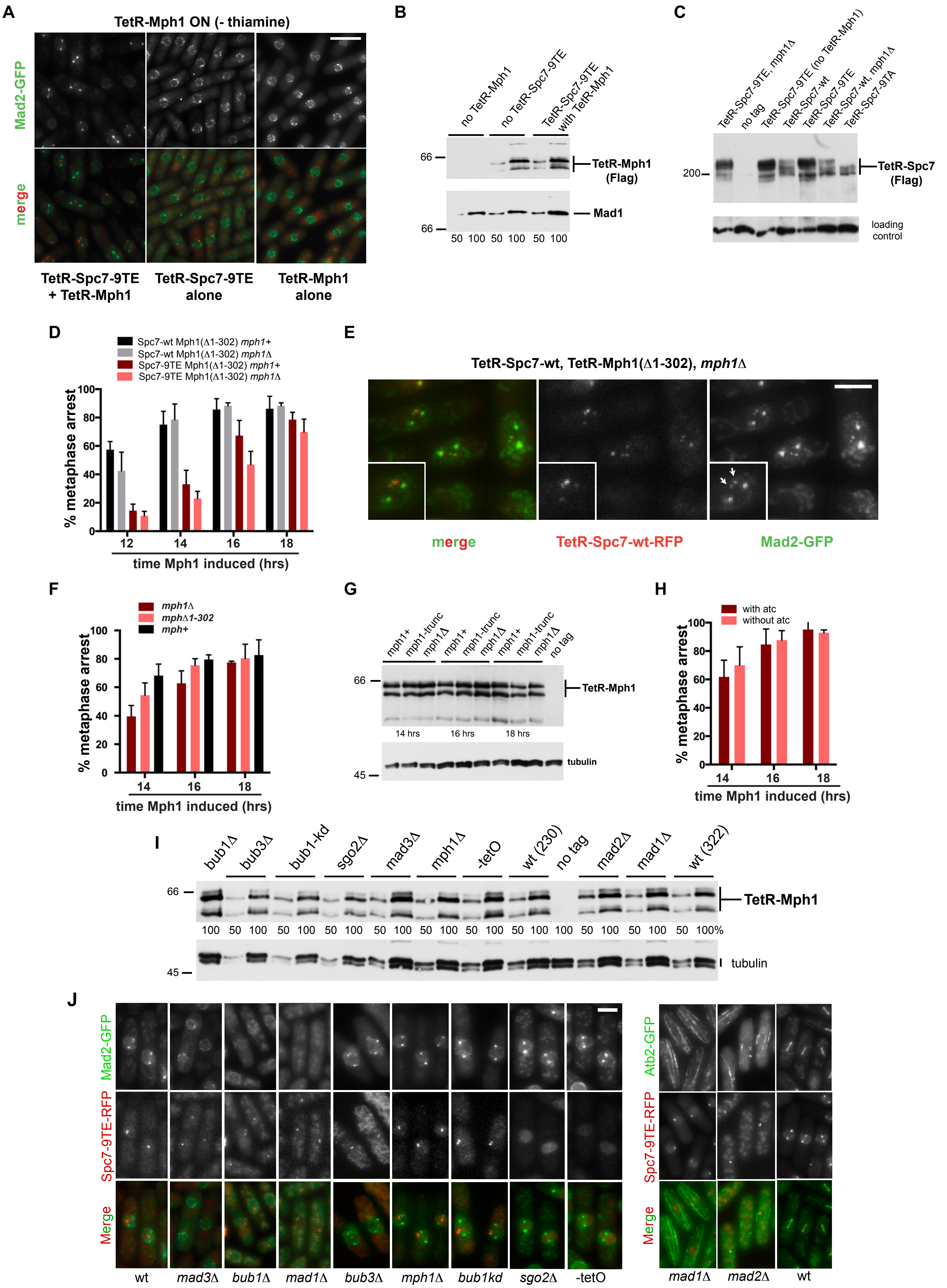
A
TetR-Mph1 ON (- thiamine)
Mad2-GFP
merge
TetR-Spc7-9TE + TetR-Mph1
TetR-Spc7-9TE alone
TetR-Mph1 alone
B
no TetR-Mph1
no TetR-Spc7-9TE
TetR-Spc7-9TE with TetR-Mph1
66
TetR-Mph1 (Flag)
Mad1
50 100 50 100 50 100
C
TetR-Spc7-9TE, mph1Δ
no tag
TetR-Spc7-9TE (no TetR-Mph1)
TetR-Spc7-wt
TetR-Spc7-9TE
TetR-Spc7-wt, mph1Δ
TetR-Spc7-9TA
200
TetR-Spc7 (Flag)
loading control
D
Spc7-wt Mph1(Δ1-302) mph1+
Spc7-wt Mph1(Δ1-302) mph1Δ
Spc7-9TE Mph1(Δ1-302) mph1+
Spc7-9TE Mph1(Δ1-302) mph1Δ
% metaphase arrest
0 20 40 60 80 100
12 14 16 18
time Mph1 induced (hrs)
E
TetR-Spc7-wt, TetR-Mph1(Δ1-302), mph1Δ
merge
TetR-Spc7-wt-RFP
Mad2-GFP
F
mph1Δ
mphΔ1-302
mph+
% metaphase arrest
0 20 40 60 80 100
14 16 18
time Mph1 induced (hrs)
G
mph1+ mph1-trunc mph1Δ mph1+ mph1-trunc mph1Δ mph1+ mph1-trunc mph1Δ no tag
66
TetR-Mph1
14 hrs 16 hrs 18 hrs
tubulin
45
H
with atc
without atc
% metaphase arrest
0 20 40 60 80 100
14 16 18
time Mph1 induced (hrs)
I
bub1Δ bub3Δ bub1-kd sgo2Δ mad3Δ mph1Δ -tetO wt (230) no tag mad2Δ mad1Δ wt (322)
66
TetR-Mph1
100 50 100 50 100 50 100 50 100 50 100 50 100 50 100 100 50 100 50 100 50 100%
tubulin
45
J
Mad2-GFP
Spc7-9TE-RFP
Merge
wt mad3Δ bub1Δ mad1Δ bub3Δ mph1Δ bub1kd sgo2Δ -tetO
Atb2-GFP
Spc7-9TE-RFP
Merge
mad1Δ mad2Δ wt

Figure S2

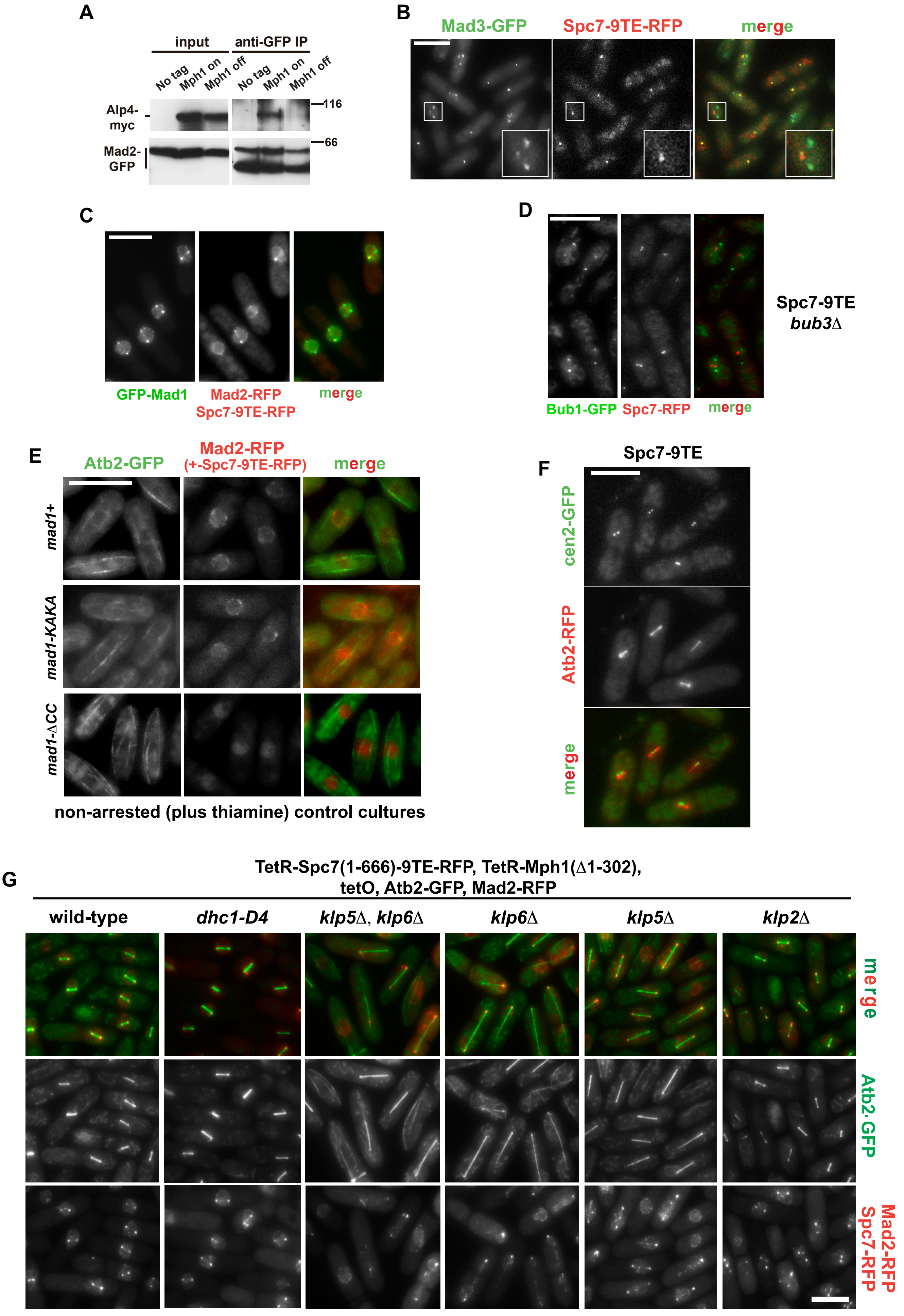

A
input
anti-GFP IP
No tag
Mph1 on
Mph1 off
No tag
Mph1 on
Mph1 off
Alp4-myc
Mad2-GFP
116
66
B
Mad3-GFP
Spc7-9TE-RFP
merge
C
GFP-Mad1
Mad2-RFP
Spc7-9TE-RFP
merge
D
Spc7-9TE
bub3Δ
Bub1-GFP
Spc7-RFP
merge
E
Atb2-GFP
Mad2-RFP
(+-Spc7-9TE-RFP)
merge
mad1+
mad1-KAKA
mad1-ΔCC
non-arrested (plus thiamine) control cultures
F
Spc7-9TE
cen2-GFP
Atb2-RFP
merge
G
TetR-Spc7(1-666)-9TE-RFP, TetR-Mph1(Δ1-302),
tetO, Atb2-GFP, Mad2-RFP
wild-type
dhc1-D4
klp5Δ, klp6Δ
klp6Δ
klp5Δ
klp2Δ
merge
Atb2-GFP
Mad2-RFP
Spc7-RFP

Figure S3

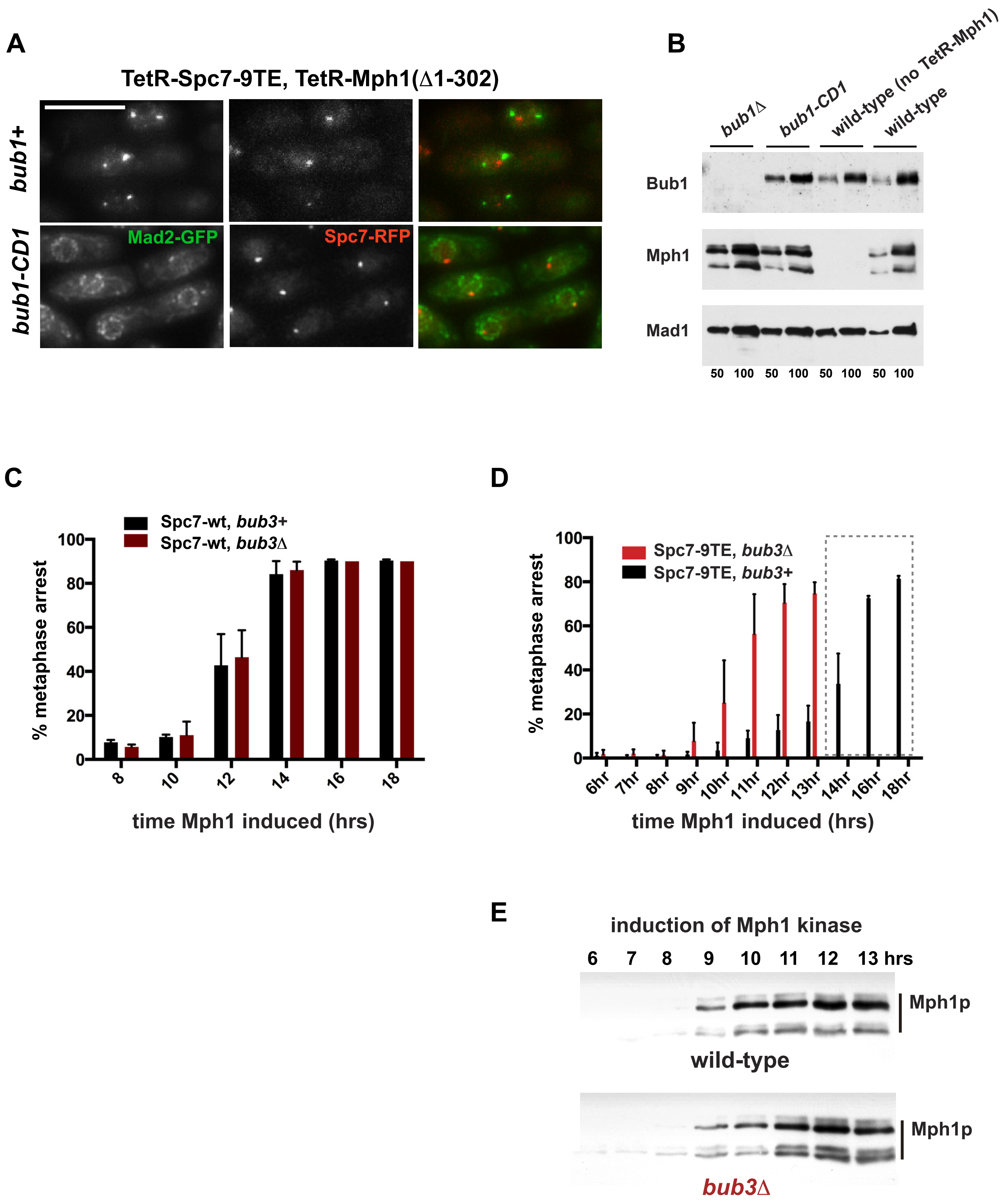
A
TetR-Spc7-9TE, TetR-Mph1(Δ1-302)
bub1+
bub1-CD1
Mad2-GFP
Spc7-RFP
B
bub1Δ
bub1-CD1
wild-type (no TetR-Mph1)
wild-type
Bub1
Mph1
Mad1
50 100 50 100 50 100 50 100
C
Spc7-wt, bub3+
Spc7-wt, bub3Δ
% metaphase arrest
100
80
60
40
20
0
8 10 12 14 16 18
time Mph1 induced (hrs)
D
Spc7-9TE, bub3Δ
Spc7-9TE, bub3+
% metaphase arrest
100
80
60
40
20
0
6hr 7hr 8hr 9hr 10hr 11hr 12hr 13hr 14hr 16hr 18hr
time Mph1 induced (hrs)
E
induction of Mph1 kinase
6 7 8 9 10 11 12 13 hrs
Mph1p
wild-type
Mph1p
bub3Δ

Figure S4

Supplemental Figure Legends

Figure S1, related to Figure 1

A) Tethered Spc7-RFP does not co-localise with the kinetochore marker Fta3-GFP.
Scale bars are 5 microns.
B) Comparison of the ability of TetR-Spc7-9TE, TetR-Spc7-wt and TetR-Spc7-9TA to recruit Bub1-GFP to the tetO array. NB. These strains all lack endogenous *mph1*.
C) Bub1-GFP dependencies: Bub1-GFP recruitment to Spc7-9TE is dependent on Bub3 but not Mad3 or Mph1.
D) Bub3-GFP dependencies: Bub3-GFP recruitment to Spc7-9TE is independent of endogenous Mph1 kinase and Mad3, but is dependent on Bub1.
E) Mad3-GFP dependencies: Mad3-GFP recruitment to Spc7-9TE is independent of endogenous Mph1 kinase, but is dependent on both Bub1 and Bub3.

Figure S2, related to Figure 2

A) Mad2-GFP was observed at spindle poles in cells that have arrested due to co-tethering of TetR-Spc7-9TE and TetR-Mph1. This was not observed in the absence of either of the Mph1 or Spc7 fusion proteins. Scale bar is 10 microns.
B) Immunoblots (anti-Flag) demonstrating similar expression of TetR-Mph1(Δ1-302) in different strains. 50 and 100 refer to 50% and 100% equivalent loading.
C) Immunoblot (anti-Flag) demonstrating the expression levels and relative gel-shifts of TetR-Spc7 constructs. TetR-Spc7-9TE has lower gel-mobility, and TetR-Spc7-9TA faster gel-mobility.
D) Comparison of the ability of TetR-Spc7-9TE and TetR-Spc7-wt to arrest cells, both with and without an endogenous copy of the *mph1+* gene. The wild-type Spc7 arrests faster than Spc7-9TE. This experiment was repeated 8 times and data plotted as mean +/- SD.
E) Imaging demonstrates that Mad2-GFP does localise to the tetO array in strains expressing TetR-Spc7-wt-RFP, TetR-Δ(1-302)Mph1. White arrows indicate Mad2-GFP enriched on the tetO array. Interestingly, this can not be detected in Spc7-9TE arrests. Scale bar is 5 microns.
F) To further analyse the impact of differing Mph1 kinase levels on signalling output, we quantitated the TetR-Spc7-9TE, TetR-Δ(1-302)Mph1 arrest in three different genetic backgrounds: with endogenous Mph1 kinase deleted; with endogenous Mph1 N-terminally truncated (diffusible); and with an endogenous wild-type gene present (likely to be kinetochore localised). Increasing the level of endogenous Mph1 kinase in this way slightly advances the time at which cells arrest. This experiment was repeated 3 times and data plotted as mean +/- SD.
G) Immunoblots (anti-Flag) demonstrating equal expression levels of the tetR-Mph1(D1-302) fusion protein in strains with different *mph1* alleles at the endogenous *Mph1+* locus.
H) Comparison of the ability of tetR-Spc7-9TE and tetR-Spc7-wt to arrest cells, with and without the presence of anhydro-tetracycline (atc) in the growth media. This experiment was repeated 10 times and data plotted as mean +/- SD. No significant effect was observed (unpaired t-test).
I) Immunoblot (anti-Flag) demonstrating equal expression levels of the tetR-Mph1(Δ1-302) fusion protein in the *mad/bub/sgo* mutant strains. All strains contain the tetO array, apart from the far right control. All strains are expressing tetR-Spc7-T9E and tetR-Mph1(Δ1-302) fusion proteins. 50 and 100 represent 50 and 100% loading of samples.
J) No arrest was observed in *mad1*Δ, *mad2*Δ, *mad3*Δ*,* or *bub1*Δ strains co-expressing tetR-Spc7-9TE and tetR-Mph1(Δ1-302), but *mph1*Δ*, bub3*Δ*, bub1-kd, or sgo2*Δ strains did arrest efficiently. Arrest was scored using either Mad2-GFP (left panels), or Atb2-GFP (three right hand panels). All of these strains contain the tetO array, apart from the control strain labelled -tetO and *sgo2*Δ. Scale bar is 5 microns.

Figure S3, related to Figure 3

All the spindle checkpoint proteins accumulate at spindle poles.
A) Mad2-GFP was co-immunoprecipitated with the gamma tubulin complex component Alp4 in Spc7-Mph1 arrested-cells. Strains were arrested for 16 hours.
B) Mad3-GFP is seen at spindle poles and at the tetO array in TetR-Spc7-Mph1 arrested cells. Scale bar is 5 microns.
C) GFP-Mad1 (note that a C-terminal tag on Mad1 is not fully functional) accumulates at the spindle poles in arrested cells. Scale bar is 10 microns.

D) Bub1-GFP accumulates at spindle poles in a Bub3-independent fashion. This is consistent with the model whereby Mad1 moves to spindle poles in a Cut7-dependent manner, and takes bound Bub1 with it. Note, if present in cells, Bub3-GFP is also taken to the poles of arrested cells (not shown). Scale bar is 10 microns.
E) These strains have not had TetR-Mph1 induced (they are growing in media containing thiamine, and so don't arrest and display interphase microtubule arrays). Mad2-GFP decorates the nuclear periphery in the *mad1-KAKA* allele, but is found to be diffuse throughout the nucleus in the *mad1-ΔCC1* allele. This allele will be described in detail in another publication (KM and KGH, in preparation). Scale bar is 10 microns.
F) In these arrested strains (containing TetR-Spc7-9TE and TetR-Mph1(Δ1-302)), cen2-GFP [S1] images demonstrate that chromosome 2 is efficiently bi-oriented on the short metaphase spindle (Atb2-RFP). Thus the synthetic arrest isn't perturbing kinetochore microtubule interactions. Scale bar is 5 microns.
G) *klp2, klp5, klp6* and dynein motor mutations[S2] did not affect the movement of Mad2-RFP to spindle poles. Scale bar is 5 microns. Note that the metaphase spindle length is significantly affected in some of these kinesin mutants, as has been previously reported [S3-S5]. Scale bar is 10 microns.

Figure S4, related to Figure 4
A) **Cells containing the *bub1-CD1* allele are unable to arrest** when co-expressing TetR-Spc7-9TE and TetR-Mph1(Δ1-302), demonstrating that the Mad1-Bub1 interaction is necessary to arrest such cells, as it is for other checkpoint arrests [S6]. Scale bar is 10 microns.
B) Immunoblots demonstrating equivalent expression of Bub1, Mph1, and Mad1 in these strains. Bub1 and Mad1 were detected with our own polyclonal antibodies (these are new Bub1 antibodies, made as previously described [S7]), and Mph1 with anti-Flag.
C) The TetR-Spc7-wt, TetR-Mph1(Δ1-302) arrest is not advanced in the absence of Bub3p. This experiment was performed 3 times and is plotted as the mean +/- SD at each time point. Note, this is unlike the effects on Spc7-9TE and Spc7-9TA arrests which were significantly advanced (Figure 4). Presumably this means that the Spc7-wt function is not rate-limiting in checkpoint arresting cells.
D) The effect of Bub3, in advancing the Spc7-9TE arrest, is also observed in the presence of endogenous Mph1 kinase. This experiment was repeated seven times and is plotted as the mean +/- SD at each time point. The later time points (within the dashed box) were usually scored from cultures that had been induced earlier.
E) Immunoblot (anti-Flag) demonstrating that Mph1 is induced with similar kinetics in strains being analysed (all were analysed, just Spc7-9TE +/- *bub3* are show here, for simplicity).

Supplemental Experimental Procedures

Construction of TetR fusion constructs

P_{nmt81}-2xFLAG-rTetR-mph1$_{\Delta 1\text{-}302}$

rTetR was amplified out from *pAK2* (gift from Alexander Kagansky, Allshire lab), digested with NheI and AseI, and inserted into a pHFF81C vector (gift from Ken Sawin) digested with NheI and NdeI. *mph1*$_{\Delta 1\text{-}302}$ was amplified from genomic DNA (strain from Silke Hauf) and inserted into tTetR-pHFF81C using Gateway recombination.

pLYS1U-P_{adh15}-NLS-2xFLAG-rTetR-spc7$_{1\text{-}666}$

pRAD15 (gift from Robin Allshire) was amplified using phosphorylated primers before DpnI digestion and re-ligation to re-create the vector with NheI and XhoI sites. These sites were then used to insert a PCR fragment *NLS-rtTA-mCherry-FLAG-ccdB* into *pRAD15*. *P_{adh15}-NLS-rtTA-mCherry-2xFLAG-ccdB* was then amplified out and subsequently joined to a PCR fragment containing the *pLYS1U* backbone (gift from Jonathan Millar) using KpnI and XhoI to form *pLYS1U-P_{adh15}-NLS-2xFLAG-rTetR-ccdB*. *spc7$_{1\text{-}666}$*(wild-type, -9TE and -9TA) were then inserted into this vector by Gateway recombination.

Construction of the Mad1 N-terminal truncation (*mad1-ΔCC*)

To truncate Mad1 expressed from its endogenous promotor, 762bp containing the promotor region, 325bp of flanking sequence upstream of this and 566bp of *mad1* coding sequence, excluding the first 500bp of *mad1*, were amplified from genomic DNA. The hygromycin resistance cassette was amplified from pFA6hphMX. The flanking sequence was digested with Kpn1 and Sal1 and cloned into pBluescript, then the resulting vector was digested with Sal1 and EcoRV and the remaining fragments were assembled by Gibson Assembly (NEB) in the following order; hygR, promotor then coding region, to give pMad1Δ1cc-hyg. To GFP tag the N-terminus of full length Mad1 the endogenous promotor was replaced by *Padh21* and *gfp* sequences inserted before 1kb of *mad1^{WT}* coding sequence as described above, using Sal1 and EcoRV digested vector and Gibson assembly. The assembled sequences were amplified by PCR and transformed into fission yeast. Standard yeast procedures, media and transformation protocols were used throughout this study.

Microscopy

For the *synthetic arrest*: cells were first grown on YES (rich) plates at 32°C overnight. The following morning, cells were transferred to 10 ml of liquid PMG medium containing 15μM thiamine and were incubated at 30°C with shaking. After seven hours, cells were harvested by spinning at 6,000 RPM for 2.5 min and washed 3 times with fresh PMG (containing supplements). The cells were transferred to fresh PMG (without thiamine) containing 10μM anhydrodoxycycline (Sigma), and then incubated at 30°C for 14, 16 and 18hrs.

Depending on the experiment the next morning the cells were harvested by spinning at 6,000 RPM for 2.5 min. The cells were washed with 1ml of clear PMG (without glucose) and harvested by spinning at 6,000 RPM for 1 min. The supernatant was removed (a small volume of media was left, depending on the pellet size) and 6-10μl of cells was deposited on a glass slide and covered with a glass coverslip. Cells were imaged immediately using a 100x oil immersion lens and a Zeiss Axiovert 200M microscope (Carl Zeiss Ltd.), equipped with a CoolSnap CCD camera (Photometrics) and Slidebook 5.0 software (3i, Intelligent Imaging Innovations, Inc.). Typical acquisition settings: 300 ms exposure (FITC & CY3), 2x binning, Z-series over 3 mm range in 0.5 mm steps (7 planes).

Crosslinking

Cells were checked under the microscope, harvested and re-suspended in reaction buffer (20mM Hepes pH7.6, 100mM KOAc). 20mM DSP (in DMSO) was added to the cells and the cross-linking reaction was left to shake slowly for 30 minutes (or 2 hours at 4°C). To quench the reaction, cells were pelleted, re-suspended in 100mM Tris pH 7.5 and left to shake for 15 minutes. Cells were pelleted at 4°C and frozen in dry ice.

Co-immunoprecipitation

The pellet was re-suspended in lysis buffer (50mM Hepes pH7.6, 75mM KCl, 1mM $MgCl_2$, 1mM EGTA, 10% Glycerol, 0.1% Triton X-100, 1mM Na_3VO_4, 10 μg/mL CLAAPE (protease inhibitor mix containing chymostatin, leupeptin, aprotinin, antipain, pepstatin, E-64 dissolved in DMSO at a concentration of 10 mg/mL), 1mM Pefabloc, 0.01mM microcystin). Zirconia beads were added to the cells and they were broken by bead-beating twice for 20 seconds and kept on ice for 30 seconds in between. Lysed cells were centrifuged at 14000 rpm for 10 minutes at 4°C to remove cell debris.

The clear lysate was incubated with anti-GFP-coupled Dynabeads for 15 minutes at 4oC. The bead were washed four times with wash buffer (50mM Hepes pH7.6, 75mM KCl, 1mM $MgCl_2$, 1mM EGTA, 10% Glycerol) and stored at -80^oC until further use. Proteins were eluted by adding sample buffer containing DTT and standing at room temperature for 15 minutes, following this, they were then run on an SDS-PAGE gel.

List of the strains used in this study

Figure 1

IY184 *lys1::P_{adh15}-rtetR-mCherry-spc7$_{1\text{-}666}$-9TE:ura4 tetO:kanR bub1-GFP mph1Δ::natR*

IY188 *lys1::P_{adh15}-rtetR-mCherry-spc7$_{1\text{-}666}$-9TE:ura4 tetO:kanR bub3-GFP mph1Δ::natR*

IY185 *lys1::P_{adh15}-rtetR-mCherry-spc7$_{1\text{-}666}$-9TE:ura4 tetO:kanR mad3-GFP mph1Δ::natR*

IL322 *lys1::P_{adh15}-rtetR-mCherry-spc7$_{1\text{-}666}$-9TE:ura4 tetO:kanR leu1$^+$:P_{nmt81}rtetR-mph1(D1-302) GFP-atb2:leu+ mad2-RFP:natR*

Figure 2

IY230 *lys1::P_{adh15}-rtetR-mCherry-spc7$_{1\text{-}666}$-9TE:ura4 tetO:kanR leu1$^+$:P_{nmt81}rtetR-mph1(D1-302) mad2-GFP:his3*

IY232 *tetO:kanR, P_{nmt81}rtetR-mph1(D1-302) mad2-GFP:his3*

IL386 *lys1::P_{adh15}-rtetR-mCherry-spc7$_{1\text{-}666}$-9TE:ura4 tetO:kanR mad2-GFP:his3*

IL787 *lys1::P_{adh15}-rtetR-mCherry-spc7$_{1\text{-}666}$:ura4 tetO:kanR leu1$^+$:P_{nmt81}rtetR-mph1(D1-302) mad2-GFP:his3*

IL673 *lys1::P_{adh15}-rtetR-mCherry-spc7$_{1\text{-}666}$-9TA:ura4 tetO:kanR leu1$^+$:P_{nmt81}rtetR-mph1(D1-302) mad2-GFP:his3*

KM1437 *lys1::P_{adh15}-rtetR-mCherry-spc7$_{1\text{-}666}$:ura4 leu1$^+$:P_{nmt81}rtetR-mph1(D1-302) mad2-GFP:his3*

IY222 *lys1::P_{adh15}-rtetR-mCherry-spc7$_{1\text{-}666}$-9TE:ura4 leu1$^+$:P_{nmt81}rtetR-mph1(D1-302) mad2-GFP:his3*

IL473 *lys1::P_{adh15}-rtetR-mCherry-spc7$_{1\text{-}666}$-9TE:ura4 tetO:kanR leu1$^+$:P_{nmt81}-mph1(D1-302) mad2-GFP:his3*

IL146 *lys1::P_{adh15}-rtetR-mCherry-spc7$_{1\text{-}666}$-9TE:ura4 tetO:kanR leu1$^+$:P_{nmt81}rtetR-mph1(D1-302) mad3Δ::Ura4+ mad2-GFP:his3*

IL 245 *lys1::P_{adh15}-rtetR-mCherry-spc7$_{1\text{-}666}$-9TE:ura4 tetO:kanR leu1$^+$:P_{nmt81}rtetR-mph1(D1-302) bub1Δ::natR mad2-GFP:his3*

IY240 *lys1::P_{adh15}-rtetR-mCherry-spc7$_{1\text{-}666}$-9TE:ura4 tetO:kanR leu1$^+$:P_{nmt81}rtetR-mph1(D1-302) bub3Δ::hygR mad2-GFP:his3*

IL133 *lys1::P_{adh15}-rtetR-mCherry-spc7$_{1\text{-}666}$-9TE:ura4 tetO:kanR leu1$^+$:P_{nmt81}rtetR-mph1(D1-302) mph1Δ::natR mad2-GFP:his3*

IL277 *lys1::P_{adh15}-rtetR-mCherry-spc7$_{1\text{-}666}$-9TE:ura4 tetO:kanR leu1$^+$:P_{nmt81}rtetR-mph1(D1-302) bub1-kd mad2-GFP:his3*

IL322 *lys1::P_{adh15}-rtetR-mCherry-spc7$_{1\text{-}666}$-9TE:ura4 tetO:kanR leu1$^+$:P_{nmt81}rtetR-mph1(D1-302) GFP-atb2:leu+ mad2-RFP:natR*

IL604 *lys1::P_{adh15}-rtetR-mCherry-spc7$_{1\text{-}666}$-9TE:ura4 tetO:kanR leu1$^+$:P_{nmt81}rtetR-mph1(D1-302) GFP-atb2:leu+ mad2Δ::ura4+*

IL609 *lys1::P_{adh15}-rtetR-mCherry-spc7$_{1\text{-}666}$-9TE:ura4 tetO:kanR leu1$^+$:P_{nmt81}rtetR-mph1(D1-302) GFP-atb2:leu+ mad1Δ::hygR mad2-RFP:natR*

SS18 *lys1::P_{adh15}-rtetR-mCherry-spc7$_{1\text{-}666}$-9TE:ura4 leu1$^+$:P_{nmt81}rtetR-mph1(D1-302) sgo2Δ::natR mad2-GFP:his3*

Figure 3

IY239 *lys1::P_{adh15}-rtetR-mCherry-spc7$_{1\text{-}666}$-9TE:ura4 tetO:kanR leu1$^+$:P_{nmt81}rtetR-mph1(D1-302) pcp1-mcherry::natR mad2-GFP:his3*

IY242 *lys1::P_{adh15}-rtetR-mCherry-spc7$_{1\text{-}666}$-9TE:ura4 tetO:kanR leu1$^+$:P_{nmt81}rtetR-mph1(D1-302) fta3-tTd:natR mad2-GFP:his3*

IL322 *lys1::P_{adh15}-rtetR-mCherry-spc7$_{1\text{-}666}$-9TE:ura4 tetO:kanR leu1$^+$:P_{nmt81}rtetR-mph1(D1-302) GFP-atb2:leu+ mad2-RFP:natR*

IL634 *lys1::P_{adh15}-rtetR-mCherry-spc7$_{1\text{-}666}$-9TE:ura4 tetO:kanR leu1$^+$:P_{nmt81}rtetR-mph1(D1-302) GFP-atb2:leu+ mad1-KAKA mad2-RFP:natR*

KM1415 lys1::P$_{adh15}$-rtetR-mCherry-spc7$_{1-666}$-9TE:ura4 tetO:kanR leu1^{+}:P$_{nmt81}$rtetR-mph1(D1-302) GFP-atb2:leu+ mad1-Δcc:hygR mad2-RFP:natR

Figure 4

IL250 lys1::P$_{adh15}$-rtetR-mCherry-spc7$_{1-666}$-9TE:ura4 tetO:kanR leu1^{+}:P$_{nmt81}$rtetR-mph1(D1-302) bub1-GFP:his3

IL615 lys1::P$_{adh15}$-rtetR-mCherry-spc7$_{1-666}$-9TE:ura4 leu1^{+}:P$_{nmt81}$rtetR-mph1(D1-302) bub1-GFP:his3

IL382 lys1::P$_{adh15}$-rtetR-mCherry-spc7$_{1-666}$-9TE:ura4 tetO:kanR leu1^{+}:P$_{nmt81}$rtetR-mph1(D1-302)

IL626 lys1::P$_{adh15}$-rtetR-mCherry-spc7$_{1-666}$-9TE:ura4 tetO:kanR leu1^{+}:P$_{nmt81}$rtetR-mph1(D1-302) bub1CD1 mad2-GFP:his3

IY230 lys1::P$_{adh15}$-rtetR-mCherry-spc7$_{1-666}$-9TE:ura4 tetO:kanR leu1^{+}:P$_{nmt81}$rtetR-mph1(D1-302) mad2-GFP:his3

IL266 lys1::P$_{adh15}$-rtetR-mCherry-spc7$_{1-666}$-9TE:ura4 tetO:kanR leu1^{+}:P$_{nmt81}$rtetR-mph1(D1-302) bub3Δ::hygR bub1-GFP:his3

IL133 lys1::P$_{adh15}$-rtetR-mCherry-spc7$_{1-666}$-9TE:ura4 tetO:kanR leu1^{+}:P$_{nmt81}$rtetR-mph1(D1-302) mph1Δ::natR mad2-GFP:his3

IL738 lys1::P$_{adh15}$-rtetR-mCherry-spc7$_{1-666}$-9TE:ura4 tetO:kanR leu1^{+}:P$_{nmt81}$rtetR-mph1(D1-302) bub3Δ::hygR mph1Δ::natR mad2-GFP:his3

IL708 lys1::P$_{adh15}$-rtetR-mCherry-spc7$_{1-666}$-9TA:ura4 tetO:kanR leu1^{+}:P$_{nmt81}$rtetR-mph1(D1-302) bub3Δ::hygR mad2-GFP:his3

IL711 lys1::P$_{adh15}$-rtetR-mCherry-spc7$_{1-666}$-9TA:ura4 tetO:kanR leu1^{+}:P$_{nmt81}$rtetR-mph1(D1-302) bub3Δ::hygR mph1Δ::natR mad2-GFP:his3

IL673 lys1::P$_{adh15}$-rtetR-mCherry-spc7$_{1-666}$-9TA:ura4 tetO:kanR leu1^{+}:P$_{nmt81}$rtetR-mph1(D1-302) mad2-GFP:his3

Supplemental Figures

Figure S1

IY170 lys1::P$_{adh15}$-rtetR-mCherry-spc7$_{1-666}$-9TE:ura4 tetO:kanR fta3-GFP:kanR

IY184 lys1::P$_{adh15}$-rtetR-mCherry-spc7$_{1-666}$-9TE:ura4 tetO:kanR bub1-GFP mph1Δ::natR

IL272 lys1::P$_{adh15}$-rtetR-mCherry-spc7$_{1-666}$-9TE:ura4 tetO:kanR bub1-GFP:his3+ mph1Δ::natR bub3Δ::hygR

IY257 lys1::P$_{adh15}$-rtetR-mCherry-spc7$_{1-666}$-9TE:ura4 tetO:kanR bub1-GFP:his3+ mph1Δ::natR mad3Δ::ura4+

IY185 lys1::P$_{adh15}$-rtetR-mCherry-spc7$_{1-666}$-9TE:ura4 tetO:kanR mad3-GFP:his3+ mph1Δ::natR

IL433 lys1::P$_{adh15}$-rtetR-mCherry-spc7$_{1-666}$-9TE:ura4 tetO:kanR mad3-GFP:his3+ bub3Δ::hygR mph1Δ::natR

IL432 lys1::P$_{adh15}$-rtetR-mCherry-spc7$_{1-666}$-9TE:ura4 tetO:kanR mad3-GFP:his3+ bub1Δ::ura4+ mph1Δ::natR

IY188 lys1::P$_{adh15}$-rtetR-mCherry-spc7$_{1-666}$-9TE:ura4 tetO:kanR bub3-GFP:his3+ mph1Δ::natR

IL342 lys1::P$_{adh15}$-rtetR-mCherry-spc7$_{1-666}$-9TE:ura4 tetO:kanR bub3-GFP:his3+ mad3Δ::ura4+ mph1Δ::natR

IL404 lys1::P$_{adh15}$-rtetR-mCherry-spc7$_{1-666}$-9TE:ura4 tetO:kanR bub3-GFP:his3+ bub1Δ::ura4+ mph1Δ::natR

IL743 lys1::P$_{adh15}$-rtetR-mCherry-spc7$_{1-666}$-9TA:ura4 tetO:kanR mph1Δ::natR bub1-GFP:his3+

IL755 lys1::P$_{adh15}$-rtetR-mCherry-spc7$_{1-666}$:ura4 tetO:kanR mph1Δ::natR bub1-GFP:his3+

Figure S2

IY230 lys1::P$_{adh15}$-rtetR-mCherry-spc7$_{1-666}$-9TE:ura4 tetO:kanR leu1^{+}:P$_{nmt81}$rtetR-mph1(D1-302) mad2-GFP:his3

IY232 tetO:kanR leu1^{+}:P$_{nmt81}$rtetR-mph1(D1-302) mad2-GFP:his3

IL386 lys1::P$_{adh15}$-rtetR-mCherry-spc7$_{1-666}$-9TE:ura4 tetO:kanR mad2-GFP:his3

IL133 lys1::P$_{adh15}$-rtetR-mCherry-spc7$_{1-666}$-9TE:ura4 tetO:kanR leu1^{+}:P$_{nmt81}$rtetR-mph1(D1-302) mph1Δ::natR mad2-GFP:his3

KM10 ade6-210 leu1-32 ura4-D18

IL787 lys1::P$_{adh15}$-rtetR-mCherry-spc7$_{1-666}$:ura4 tetO:kanR leu1^{+}:P$_{nmt81}$rtetR-mph1(D1-302) mad2-GFP:his3

IL673 lys1::P$_{adh15}$-rtetR-mCherry-spc7$_{1-666}$-9TA:ura4 tetO:kanR leu1^{+}:P$_{nmt81}$rtetR-mph1(D1-302) mad2-GFP:his3

IL788 lys1::P$_{adh15}$-rtetR-mCherry-spc7$_{1-666}$:ura4 tetO:kanR leu1^{+}:P$_{nmt81}$rtetR-mph1(D1-302) mph1Δ::natR mad2-GFP:his3

IY222 lys1::P$_{adh15}$-rtetR-mCherry-spc7$_{1-666}$-9TE:ura4 leu1^{+}:P$_{nmt81}$rtetR-mph1(D1-302) mad2-GFP:his3

IL322 lys1::P$_{adh15}$-rtetR-mCherry-spc7$_{1-666}$-9TE:ura4 tetO:kanR leu1^{+}:P$_{nmt81}$rtetR-mph1(D1-302) GFP-atb2:leu+ mad2-RFP:natR

IL619 *lys1::P_{adh15}-rtetR-mCherry-spc7$_{1-666}$-9TE:ura4 tetO:kanR leu1^{+}:P_{nmt81}rtetR-mph1(D1-302) mph1(D1-302)-GFP:G418, GFP-atb2:leu+ mad2-RFP:natR*

IL665 *lys1::P_{adh15}-rtetR-mCherry-spc7$_{1-666}$-9TE:ura4 tetO:kanR leu1^{+}:P_{nmt81}rtetR-mph1(D1-302) mph1Δ::natR, GFP-atb2:leu+ mad2-RFP:natR*

IL146 *lys1::P_{adh15}-rtetR-mCherry-spc7$_{1-666}$-9TE:ura4 tetO:kanR leu1^{+}:P_{nmt81}rtetR-mph1(D1-302) mad3Δ::ura4+ mad2-GFP:his3*

IL 245 *lys1::P_{adh15}-rtetR-mCherry-spc7$_{1-666}$-9TE:ura4 tetO:kanR leu1^{+}:P_{nmt81}rtetR-mph1(D1-302) bub1Δ::natR mad2-GFP:his3*

IY240 *lys1::P_{adh15}-rtetR-mCherry-spc7$_{1-666}$-9TE:ura4 tetO:kanR leu1^{+}:P_{nmt81}rtetR-mph1(D1-302) bub3Δ::hygR mad2-GFP:his3*

IL133 *lys1::P_{adh15}-rtetR-mCherry-spc7$_{1-666}$-9TE:ura4 tetO:kanR leu1^{+}:P_{nmt81}rtetR-mph1(D1-302) mph1Δ::natR mad2-GFP:his3*

IL277 *lys1::P_{adh15}-rtetR-mCherry-spc7$_{1-666}$-9TE:ura4 tetO:kanR leu1^{+}:P_{nmt81}rtetR-mph1(D1-302) bub1-kd mad2-GFP:his3*

IL322 *lys1::P_{adh15}-rtetR-mCherry-spc7$_{1-666}$-9TE:ura4 tetO:kanR leu1^{+}:P_{nmt81}rtetR-mph1(D1-302) GFP-atb2:leu+ mad2-RFP:natR*

IL604 *lys1::P_{adh15}-rtetR-mCherry-spc7$_{1-666}$-9TE:ura4 tetO:kanR leu1^{+}:P_{nmt81}rtetR-mph1(D1-302) GFP-atb2:leu+ mad2Δ::ura4+*

IL609 *lys1::P_{adh15}-rtetR-mCherry-spc7$_{1-666}$-9TE:ura4 tetO:kanR leu1^{+}:P_{nmt81}rtetR-mph1(D1-302) GFP-atb2:leu+ mad1Δ::hygR mad2-RFP:natR*

SS18 *lys1::P_{adh15}-rtetR-mCherry-spc7$_{1-666}$-9TE:ura4 leu1^{+}:P_{nmt81}rtetR-mph1(D1-302) sgo2Δ::natR mad2-GFP:his3*

Figure S3

IL511 *lys1::P_{adh15}-rtetR- mCherry-spc7$_{1-666}$-9TE:ura4 tetO:kanR leu1^{+}:P_{nmt81}rtetR-mph1(D1-302) alp4-myc:kanR mad2-GFP:his3*

KM10 *ade6-210 leu1-32 ura4-D18*

IY235 *lys1::P_{adh15}-rtetR-mCherry-spc7$_{1-666}$-9TE:ura4 tetO:kanR leu1::P_{nmt81}rtetR-mph1(D1-302) Mad3-GFP:his3*

IL266 *lys1::P_{adh15}-rtetR-mCherry-spc7$_{1-666}$-9TE:ura4 tetO:kanR leu1^{+}:P_{nmt81}rtetR-mph1(D1-302) Bub1-GFP:his3 bub3Δ::hygR*

KM1440 *lys1::P_{adh15}-rtetR-mCherry-spc7$_{1-666}$-9TE:ura4 leu1^{+}::tetO:kanR leu1^{+}:P_{nmt81}rtetR-mph1(D1-302) mad2-RFP::natR P_{adh21}-mad1-GFP::hygR*

IL322 *lys1::P_{adh15}-rtetR-mCherry-spc7$_{1-666}$-9TE:ura4 tetO:kanR leu1^{+}:P_{nmt81}rtetR-mph1(D1-302) GFP-atb2:leu+ mad2-RFP:natR*

KM1415 *lys1::P_{adh15}-rtetR-mCherry-spc7$_{1-666}$-9TE:ura4 tetO:kanR leu1^{+}::P_{nmt81}rtetR-mph1(D1-302) GFP-atb2:leu+ mad1-Δcc:hygR mad2-RFP:natR*

IL634 *lys1::P_{adh15}-rtetR-mCherry-spc7$_{1-666}$-9TE:ura4 tetO:kanR leu1^{+}:P_{nmt81}rtetR-mph1(D1-302) GFP-atb2:leu+ mad1-KAKA mad2-RFP:natR*

IL558 lys1::P_{adh15}-rtetR-mCherry-spc7$_{1-666}$-9TE:ura4 tetO:kanR leu1^{+}:P_{nmt81}rtetR-mph1(D1-302) GFP-atb2:leu+ klp5Δ::ura4+ mad2-RFP:natR

IL560 lys1::P_{adh15}-rtetR-mCherry-spc7$_{1-666}$-9TE:ura4 tetO:kanR leu1^{+}:P_{nmt81}rtetR-mph1(D1-302) GFP-atb2:leu+ klp6Δ::ura4+ mad2-RFP:natR

IL664 lys1::P_{adh15}-rtetR-mCherry-spc7$_{1-666}$-9TE:ura4 tetO:kanR leu1^{+}:P_{nmt81}rtetR-mph1(D1-302) GFP-atb2:leu+ klp6Δ::ura4+ klp5Δ::ura4+ mad2-RFP:natR

IL 540 *lys1::P_{adh15}-rtetR-mCherry-spc7$_{1-666}$-9TE:ura4 tetO:kanR leu1^{+}:P_{nmt81}rtetR-mph1(D1-302) GFP-atb2:leu+ klp2Δ::hygR mad2-RFP:natR*

IL 672 *lys1::P_{adh15}-rtetR-mCherry-spc7$_{1-666}$-9TE:ura4 tetO:kanR leu1^{+}:P_{nmt81}rtetR-mph1(D1-302) GFP-atb2:leu+ dhc1-D4::ura4+ mad2-RFP:natR*

SS19 *lys1::P_{adh15}-rtetR-mCherry-spc7$_{1-666}$-9TE:ura4 leu1^{+}:P_{nmt81}rtetR-mph1(D1-302) cen2::kanR:ura4+:lacO his7+::lacI-GFP P_{adh15}:RFP-atb2:natR*

Figure S4

IL245 *lys1::P_{adh15}-rtetR-mCherry-spc7$_{1\text{-}666}$-9TE:ura4 tetO:kanR leu1^{+}:P_{nmt81}rtetR-mph1(D1-302) bub1Δ::natR mad2-GFP:his3*

IL626 *lys1::P_{adh15}-rtetR-mCherry-spc7$_{1\text{-}666}$-9TE:ura4 tetO:kanR leu1^{+}:P_{nmt81}rtetR-mph1(D1-302) bub1CD1 mad2-GFP:his3*

IL386 *lys1::P_{adh15}-rtetR-mCherry-spc7$_{1\text{-}666}$-9TE:ura4 tetO:kanR mad2-GFP:his3*

KM10 *ade6-210 leu1-32 ura4-D18*

IY230 *lys1::P_{adh15}-rtetR-mCherry-spc7$_{1\text{-}666}$-9TE:ura4 tetO:kanR leu1^{+}:P_{nmt81}rtetR-mph1(D1-302) mad2-GFP:his3*

IY240 *lys1::P_{adh15}-rtetR-mCherry-spc7$_{1\text{-}666}$-9TE:ura4 tetO:kanR leu1^{+}:P_{nmt81}rtetR-mph1(D1-302) bub3Δ::hygR mad2-GFP:his3*

IL788 *lys1::P_{adh15}-rtetR-mCherry-spc7$_{1\text{-}666}$:ura4 tetO:kanR leu1^{+}:P_{nmt81}rtetR-mph1(D1-302) mph1Δ::natR mad2-GFP:his3*

IL719 *lys1::P_{adh15}-rtetR-mCherry-spc7$_{1\text{-}666}$:ura4 tetO:kanR leu1^{+}:P_{nmt81}rtetR-mph1(D1-302) mph1Δ::natR bub3Δ::hygR mad2-GFP:his3*

Supplemental references:

S1. Ding, D.Q., Yamamoto, A., Haraguchi, T., and Hiraoka, Y. (2004). Dynamics of homologous chromosome pairing during meiotic prophase in fission yeast. Dev Cell *6*, 329-341.

S2. Yamamoto, A., West, R.R., McIntosh, J.R., and Hiraoka, Y. (1999). A cytoplasmic dynein heavy chain is required for oscillatory nuclear movement of meiotic prophase and efficient meiotic recombination in fission yeast. J Cell Biol *145*, 1233-1249.

S3. West, R.R., Malmstrom, T., Troxell, C.L., and McIntosh, J.R. (2001). Two related kinesins, klp5+ and klp6+, foster microtubule disassembly and are required for meiosis in fission yeast. Mol Biol Cell *12*, 3919-3932.

S4. West, R.R., Malmstrom, T., and McIntosh, J.R. (2002). Kinesins klp5(+) and klp6(+) are required for normal chromosome movement in mitosis. J Cell Sci *115*, 931-940.

S5. Garcia, M.A., Koonrugsa, N., and Toda, T. (2002). Spindle-kinetochore attachment requires the combined action of Kin I-like Klp5/6 and Alp14/Dis1-MAPs in fission yeast. EMBO J *21*, 6015-6024.

S6. Heinrich, S., Sewart, K., Windecker, H., Langegger, M., Schmidt, N., Hustedt, N., and Hauf, S. (2014). Mad1 contribution to spindle assembly checkpoint signalling goes beyond presenting Mad2 at kinetochores. EMBO Rep *15*, 291-298.

S7. Vanoosthuyse, V., Valsdottir, R., Javerzat, J.P., and Hardwick, K.G. (2004). Kinetochore targeting of fission yeast Mad and Bub proteins is essential for spindle checkpoint function but not for all chromosome segregation roles of Bub1p. Mol Cell Biol *24*, 9786-9801.
