# Supplementary material for: Generation of a Spindle Checkpoint Arrest from Synthetic Signaling Assemblies
Source: Curr Biol. 2017 Jan 9;27(1):137–43. doi: 10.1016/j.cub.2016.11.014 (PMC5226922; doi:10.1016/j.cub.2016.11.014)
Supplement: Document S2. Article plus Supplemental Information [file mmc2.pdf]

Report

Current Biology

Generation of a Spindle Checkpoint Arrest from Synthetic Signaling Assemblies

Graphical Abstract

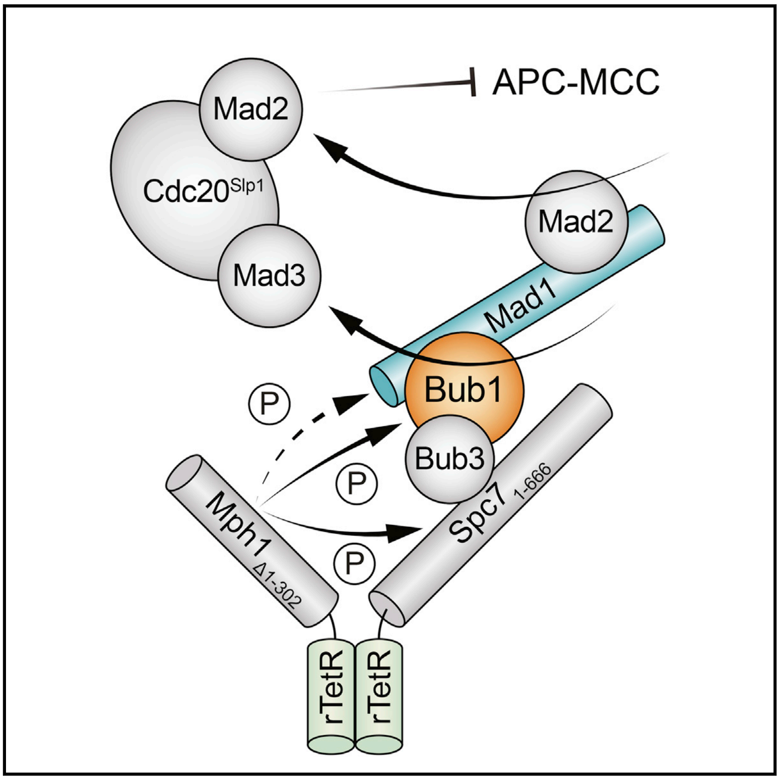

Highlights

- Synthetic signaling scaffolds generate a spindle checkpoint arrest
- The combination of KNL1 Spc7 and Mps1 Mph1 kinase generates a robust arrest
- Kinetochore, spindle, and nuclear envelope enrichment of the scaffold is not required
- Bub3 acts to inhibit premature checkpoint activation

Authors

Ivan Yuan, Ioanna Leontiou, Priya Amin, Karen M. May, Sadhbh Soper Ní Chafraidh, Eliška Zlámalová, Kevin G. Hardwick

Correspondence

kevin.hardwick@ed.ac.uk

In Brief

Yuan et al. employ synthetic protein assemblies to generate a robust spindle checkpoint arrest in fission yeast. Formation of artificial heterodimers between a checkpoint kinase and one of its key substrates is shown to be sufficient for mitotic arrest, entirely independently of the location of this complex in the yeast nucleus.

Yuan et al., 2017, Current Biology *27*, 137–143
January 9, 2017 © 2017 The Author(s). Published by Elsevier Ltd.
http://dx.doi.org/10.1016/j.cub.2016.11.014

Current Biology
Report

CellPress

Generation of a Spindle Checkpoint Arrest from Synthetic Signaling Assemblies

Ivan Yuan,[1,2] Ioanna Leontiou,[1,2] Priya Amin,[1] Karen M. May,[1] Sadhbh Soper Ní Chafraidh,[1] Eliška Zlámalová,[1] and Kevin G. Hardwick[1,3,*]
[1]Wellcome Trust Centre for Cell Biology, University of Edinburgh King's Buildings, Max Born Crescent, Edinburgh EH9 3BF, UK
[2]Co-first author
[3]Lead Contact
*Correspondence: kevin.hardwick@ed.ac.uk
http://dx.doi.org/10.1016/j.cub.2016.11.014

SUMMARY

The spindle checkpoint acts as a mitotic surveillance system, monitoring interactions between kinetochores and spindle microtubules and ensuring high-fidelity chromosome segregation [1–3]. The checkpoint is activated by unattached kinetochores, and Mps1 kinase phosphorylates KNL1 on conserved MELT motifs to generate a binding site for the Bub3-Bub1 complex [4–7]. This leads to dynamic kinetochore recruitment of Mad proteins [8, 9], a conformational change in Mad2 [10–12], and formation of the mitotic checkpoint complex (MCC: Cdc20-Mad3-Mad2 [13–15]). MCC formation inhibits the anaphase-promoting complex/cyclosome (Cdc20-APC/C), thereby preventing the proteolytic destruction of securin and cyclin and delaying anaphase onset. What happens at kinetochores after Mps1-dependent Bub3-Bub1 recruitment remains mechanistically unclear, and it is not known whether kinetochore proteins other than KNL1 have significant roles to play in checkpoint signaling and MCC generation. Here, we take a reductionist approach, avoiding the complexities of kinetochores, and demonstrate that co-recruitment of KNL1^{Spc7} and Mps1^{Mph1} is sufficient to generate a robust checkpoint signal and prolonged mitotic arrest. We demonstrate that a Mad1-Bub1 complex is formed during synthetic checkpoint signaling. Analysis of *bub3Δ* mutants demonstrates that Bub3 acts to suppress premature checkpoint signaling. This synthetic system will enable detailed, mechanistic dissection of MCC generation and checkpoint silencing. After analyzing several mutants that affect localization of checkpoint complexes, we conclude that spindle checkpoint arrest can be independent of their kinetochore, spindle pole, and nuclear envelope localization.

RESULTS AND DISCUSSION

Genetic and proteomic approaches have revealed that kinetochores are highly complex molecular machines (with ~100 kinetochore components in vertebrates [16] and ~50 in yeast [17]) and that there are approximately ten components of the spindle checkpoint machinery [2]. Amidst such complexity, separating kinetochore bi-orientation, error-correction, and microtubule attachment functions from true checkpoint activation and signaling functions is problematic. Kinetochore tethering of, e.g., Mph1-Ndc80 and Mis12-Mad1 can initiate checkpoint arrests [18, 19]. However, it is very likely that in such experiments endogenous kinetochore function is being perturbed and that these perturbations then activate the spindle checkpoint, making interpretation of the experiments complicated and rather unsatisfactory. To improve on this tethering strategy, we set out to generate a spindle checkpoint arrest from a site quite distinct from an unattached kinetochore. We employed a fission yeast strain with 112 tandem repeats of the tet operator (tetO) integrated on the arm of chromosome 1 (at the *arg3* locus, see Figure 1A). This is ~1.5 Mb away from cen1 and can thus be imaged as a distinct spot in live fission yeast cells (see Figure S1A). When we expressed the phosphomimic mutant Spc7(1-666)-9TE fused to the Tet repressor in these cells it resulted in constitutive recruitment of Bub1, Bub3, and Mad3 to the tetO array, throughout the cell cycle and independently of Mph1 kinase (Figure 1B). Note, this fusion protein only contains the first half of Spc7 (1-666) and so completely lacks its C-terminal kinetochore targeting domain. Expression of TetR-Spc7-9TA failed to recruit checkpoint proteins to the tetO array (see Figure S1B), whereas wild-type TetR-Spc7 was able to recruit Bub1, Bub3, and Mad3 but at much lower levels than TetR-Spc7-9TE and in a way that was dependent on endogenous Mph1 kinase action (see Figure S1B). This demonstrates that the "activated" Spc7-9TE binding platform is sufficient to recruit these three checkpoint proteins constitutively, and that this works ectopically and thus does not require additional kinetochore factors. Bub1p, Bub3p, and Mad3p are recruited to the array with the expected dependencies (see Figures S1C–S1E): thus, we believe that this Spc7-Bub-Mad3 complex likely acts as an independent signaling module (Figure 1C).

Co-tethering KNL1^{Spc7} and Mps1^{Mph1} Kinase Generates a Robust Mitotic Arrest

At unattached kinetochores, Bub1 is thought to recruit Mad1 [20]. However, when we expressed TetR-Spc7-9TE no detectable Mad1-Mad2 proteins were recruited to the array, and no cell-cycle delay was observed (data not shown). When we co-expressed TetR-Mad1 with TetR-Spc7-9TE, again no cell-cycle

This is an open access article under the CC BY license (http://creativecommons.org/licenses/by/4.0/).

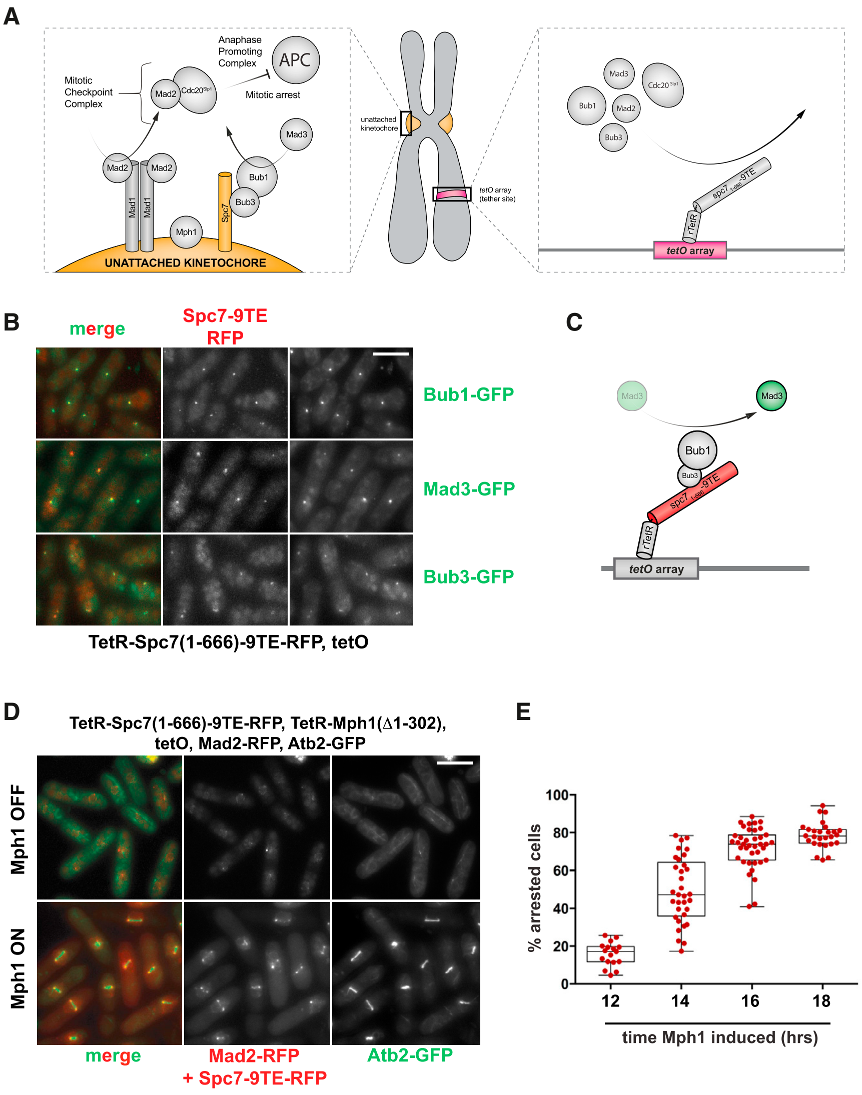

Figure 1. Co-tethering of Spc7-9TE and TetR-Mph1ΔN Generates a Robust Checkpoint Arrest

(A) Schematic model of kinetochore-based checkpoint signaling versus the synthetic tetO platform.

(B) TetR-Spc7-9TE is sufficient to recruit Bub1-GFP, Bub3-GFP, and Mad3-GFP to an array of Tet operators on a chromosome arm. Scale bar, 10 μm. See Figure S1 for TetR-Spc7wt and TetR-Spc7-9TA images.

(C) Schematic summary of Spc7-9TE tethering.

(D) Co-expression of TetR-Spc7-9TE and TetR-Mph1ΔN produces a robust mitotic arrest with short metaphase spindles. Scale bar, 10 μm.

(E) Quantitation of arrested cells after 12, 14, 16, and 18 hr of Mph1 induction (cells grown without thiamine). The plus thiamine control culture does not arrest, containing just a few mitotic cells. 36 experiments were performed and data points are plotted along with the mean and SD.

See also Figure S1.

effects were observed (data not shown). This suggests that co-recruitment of Mad1 and Bub1 is not sufficient for checkpoint signaling at least on this tetO platform. We thought that this might be because the synthetic signaling scaffold assembled there (Spc7-Bub-Mad) lacked Mph1 kinase. Therefore, instead of Mad1, we co-expressed TetR-Spc7-9TE with TetR-Δ(1-302) Mph1, being very careful not to overexpress Mph1 kinase. We particularly wanted to avoid activating the checkpoint from kinetochores, and so the N terminus of Mph1 was removed to prevent this kinase from targeting to endogenous kinetochores [21] where it might be activated and could then recruit other checkpoint complexes. Figures 1D and 1E show a very striking result: co-expression of TetR-Spc7-9TE with TetR-Δ(1-302)Mph1 was sufficient to arrest cells in mitosis. This is seen very clearly in Figure 1D where we used GFP-labeled tubulin to image the short metaphase spindles in arrested cells. Figure 1E shows that, after 16 hr of Mph1 induction, we typically see ~80% metaphase cells (cf. ~5% in strains not inducing Mph1, +thiamine). When we imaged Mad2-GFP/RFP in the arrested cells, we saw that, rather than accumulate at the tetO array with the Bub proteins, Mad2-GFP accumulated at the poles of the metaphase spindles (Figure 2A, and see Figure 3A for co-localization). Importantly, this arrest requires co-expression of both TetR-Spc7-9TE and TetR-Δ(1-302)Mph1: neither alone is sufficient for an arrest (Figures 2B and S2A–S2D), and their arrest does not require endogenous Mph1 kinase (Figures S2D–S2G).

Next, we analyzed Spc7-wt and Spc7-9TA: while Spc7-9TA had little effect on the cell cycle, we were surprised to find that Spc7-wt arrested significantly faster than Spc7-9TE (Figure 2C), with ~60% mitotic arrest after 12 hr compared to 16 hr for Spc7-9TE. To analyze this in more detail, we compared Spc7-wt and Spct-9TE arrests in strains expressing TetR-Δ(1-302)Mph1 both with and without endogenous Mph1 kinase. Figure S2D confirms that the wild-type form of this signaling scaffold is more efficient than the Spc7-9TE phosphomimic at generating a checkpoint signal. There are several possible reasons for this: perhaps the nine glutamic acid residues do not fully mimic phosphorylation, or perhaps having all nine sites modified on a single molecule is not optimal for scaffolding function (see Mad2 recruitment below).

Next, we wanted to test what level of co-enrichment of TetR-Spc7-9TE and TetR-Δ(1-302)Mph1 was necessary for initiation of an arrest (each yeast kinetochore is thought to have approximately five molecules of Spc7 [22]), and so we modified our strains by reducing the number of tet operators present, and thus the number of Spc7 and Mph1 binding sites. Strains containing four tandem copies of tetO arrested well (data not shown) and to our surprise so did strains without any tetO sequences at all (Figures 2D and S2J). Consistent with this observation, we found that addition of anhydro-tetracycline (aTc), which enhances TetR binding to the tetO array in this system, had no

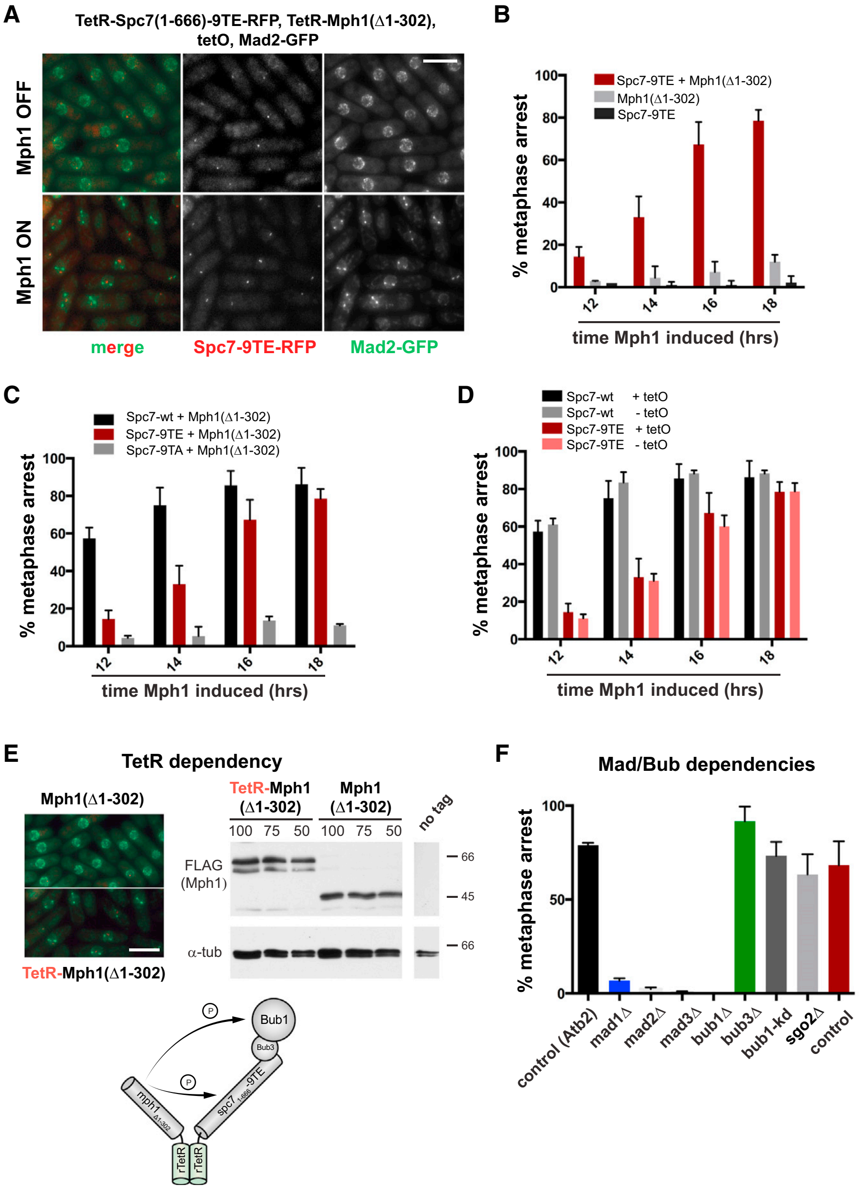

Figure 2. Dependencies for Synthetic Checkpoint Arrest

(A) Co-expression of TetR-Spc7-9TE and TetR-Mph1ΔN leads to a metaphase arrest with Mad2-GFP accumulating at the spindle poles (analyzed in detail in Figure 3). Scale bar, 10 μm.

(B) Expression of either TetR-Spc7-9TE or TetR-Mph1ΔN alone is not sufficient for robust arrest. This experiment was repeated three times and is plotted as the mean ± SD.

(C) Comparison of TetR-Spc7-9TE, TetR-wild-type Spc7 (Spc7-wt), and TetR-Spc7-9TA. The latter is unable to arrest cells, whereas the wild-type protein arrests better than Spc7-9TE. This experiment was repeated three times and is plotted as the mean ± SD.

(D) The tetO array is not necessary for Mad2-GFP accumulation at spindle poles or metaphase arrest. The mitotic arrest, for both TetR-Spc7-wt and TetR-Spc7-9TE, was compared in strains containing either 112xtetO or no tet operators. This experiment was repeated three times and is plotted as the mean ± SD.

(E) No arrest was observed when TetR was removed from the Mph1 fusion protein (Mad2-GFP did not accumulate at spindle poles). Scale bar, 10 μm. Anti-Flag (Mph1) immunoblot of whole cell extracts demonstrates that similar levels of Mph1 were expressed with and without TetR.

(F) The mitotic arrest is Mad1, Mad2, Mad3, and Bub1 dependent, but independent of Bub3, Bub1 kinase activity, and Sgo2. These strains were analyzed at least three times and data plotted as the mean ± SD. The control strain (TetR-Spc7-9TE) on the left has Atb2-GFP as reporter and on the right Mad2-RFP. All strains contained the tetO array, apart from *sgo2*Δ and its corresponding control strain. Representative images are presented in Figure S2J.

See also Figure S2.

significant effect on this arrest (Figure S2H). Our interpretation is that soluble, heterodimeric complexes formed between TetR-Spc7 and TetR-Mph1 in the nucleoplasm are sufficient for checkpoint activation. To test this directly, we removed TetR from the Mph1 construct: the resulting strains no longer arrest, and Mad2-GFP does not accumulate at spindle poles (Figure 2E). We conclude that forced interaction of these two critical upstream checkpoint components is sufficient for activation of the spindle checkpoint, and that their enrichment at the tetO array is unnecessary for these signals to induce a metaphase arrest.

If these arrests reflect a normal mode of checkpoint signaling, they should be dependent on downstream checkpoint components. We tested the dependence of this metaphase arrest on the Mad/Bub proteins and found that it required Mad1, Mad2, Mad3, and Bub1. Importantly it did not require "upstream" kinetochore-based signaling: the arrest was independent of endogenous Mph1 and Bub1 kinase activities, of Sgo2, and of Bub3 (Figures 2F and S2I–S2J). The latter is not surprising, as Bub3 is known to be unnecessary for fission yeast spindle checkpoint arrests [23, 24].

Arrested Cells Accumulate Several Checkpoint Proteins at Their Spindle Poles

Co-expression of Mps1 kinase and an Spc105 fragment has previously been demonstrated to induce a cell-cycle delay in budding yeast [25]. In that study, the rapamycin-induced heterodimers (of Mps1-Spc105) usually became enriched at endogenous kinetochores, which could then serve as a platform to generate or amplify the checkpoint signal. Some evidence was presented suggesting that the cell-cycle delay could be generated independently of kinetochores, using the *ndc10-1* mutation where kinetochores are thought to be destroyed at the restrictive temperature. The possible role of endogenous kinetochores is an important issue, and one we were keen to avoid in our system: our Mph1 construct lacks the N-terminal 302 residue kinetochore

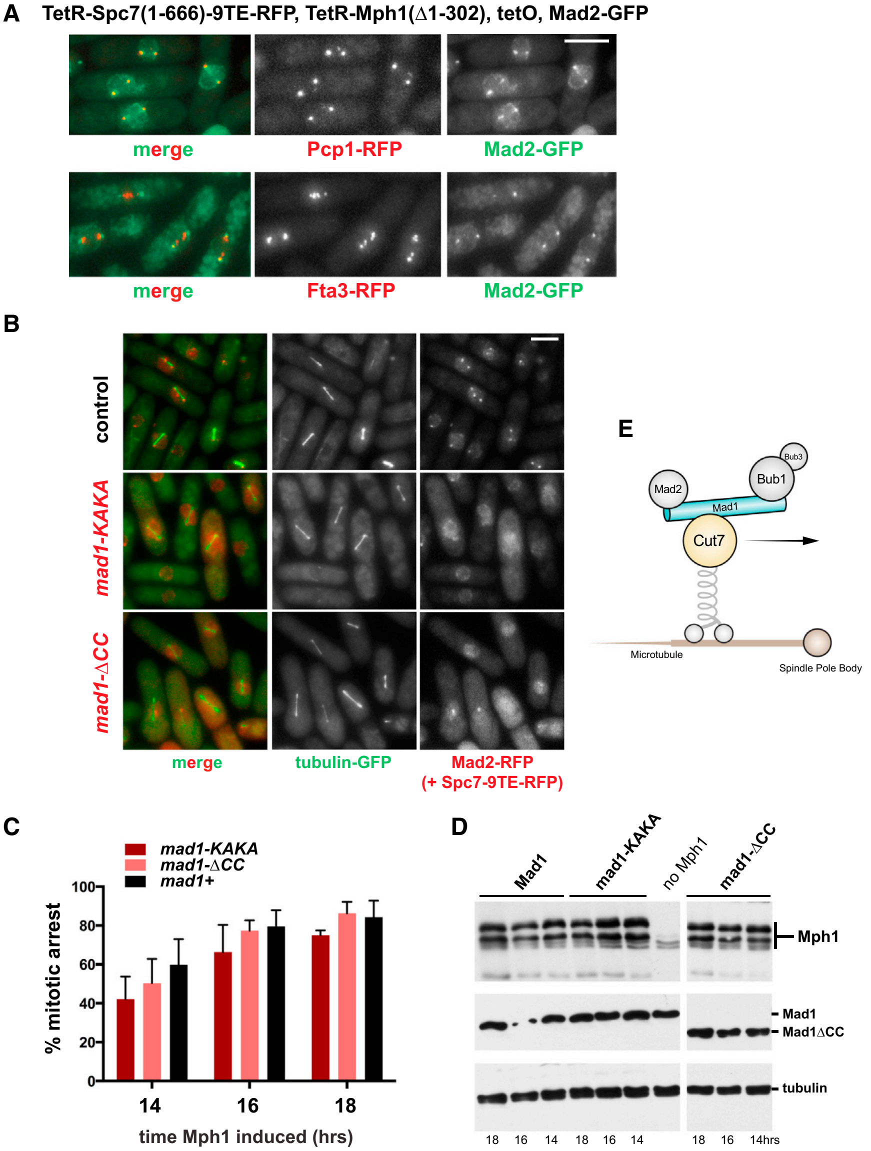

Figure 3. Mad2p Accumulates at Spindle Poles in the Synthetic Arrest, but This Is Not Necessary for the Arrest

(A) Cells were arrested with co-expression of TetR-Mph1ΔN and TetR-Spc7-9TE. Co-localization of the spindle pole marker Pcp1-RFP and Mad2-GFP is observed. The Mad2-GFP does not co-localize well with the kinetochore marker Fta3-RFP in arrested cells, although in a few cases kinetochores are close to the poles. Scale bar, 5 μm. Figure S3A demonstrates co-immunoprecipitation of Mad2 with gamma tubulin complex proteins.

(B) Strains co-expressing Spc7 and Mph1 do not accumulate Mad2-GFP at spindle poles in strains containing the *mad1-KAKA* mutation that disrupts the Mad1-Cut7 kinesin motor interaction. Other motor mutants were analyzed (*dynein*, *klp2*Δ, *klp5/6*Δ) but found to have no effect on the arrest or Mad2-GFP localization to spindle poles (see Figure S3G). Scale bar, 10 μm.

The *mad1-*Δ*CC* allele still arrests even though localization of Mad1 and Mad2 to the nuclear periphery/envelope and spindle poles is lost. This N-terminal coiled-coil domain also includes the Cut7 interaction site.

(C) Quantitation of the *mad1-KAKA* and *mad1-*Δ*CC* mutant arrests. This experiment was repeated five times and data plotted as the mean ± SD.

(D) The levels of Mph1 expression and Mad1 protein stability are not affected in these *mad1* mutants. Time of Mph1 induction (after thiamine wash-out) is indicated.

(E) Model with the Cut7 kinesin moving the Mad-Bub complex to spindle poles. This predicts that the movement of Bub1 to spindle poles is Bub3 independent, which was found to be the case (see Figure S3D).

See also Figure S3.

targeting domain, our Spc7 construct lacks the C-terminal half of the protein that targets it to kinetochores, and most of our strains lack endogenous Mph1 kinase, thereby preventing all the Mad/Bub proteins from being recruited to endogenous kinetochores [21]. We carried out co-localization experiments with kinetochore (Fta3 [26]) and spindle pole (Pcp1 [27]) markers in our arrested fission yeast cells. Figure 3A demonstrates that Mad2-GFP was not recruited to endogenous fission yeast kinetochores but instead overlapped well with gamma-tubulin and spindle pole body markers. Mad1 and Mad2 proteins have been observed at spindle poles previously, and direct interaction with the gamma-tubulin protein Alp4 and Mad2 has been described in fission yeast cells late in mitosis (post-metaphase), but its roles there remain unclear [28]. Co-immunoprecipitation confirms that Mad2-GFP interacts with Alp4 in these synthetically arrested cells (see Figure S3A). We analyzed which other checkpoint proteins were enriched at spindle poles in the arrested cells, by crossing in GFP-tagged forms of Mad1, Mad3, Bub3, and Bub1 (Figures S3B–S3D). Mad3-GFP and Bub1-GFP were recruited both to the tetO array and to spindle poles in cells co-expressing TetR-Spc7-9TE with TetR-Δ(1-310)Mph1 (see Figures S3B–S3D), as is Bub3-GFP (data not shown). Interestingly, Bub1-GFP recruitment to spindle poles did not require Bub3. Similar observations were made with TetR-Spc7-wt experiments with one important exception: in cells co-expressing TetR-Spc7-wt with TetR-Δ(1-310)Mph1, we could also detect Mad2-GFP on the tetO array (Figure S2E). This interesting observation might explain why these cells arrest faster than Spc7-9TE, as it suggests that the Mad1-Mad2 complex associates more stably with the TetR-Spc7-wt platform than with TetR-Spc7-9TE and that this stable complex may then be better able to generate the mitotic checkpoint complex (MCC) and inhibit the anaphase-promoting complex/cyclosome (APC/C).

Spindle Pole Localization Is Not Necessary for Checkpoint Arrest

We wanted to test whether the spindle pole localization was relevant to generation of the checkpoint arrest in these cells. In

human cells, checkpoint proteins are stripped from the outer kinetochore upon microtubule attachment and transported to spindle poles in a dynein-dependent fashion [29]. This is thought to be one way vertebrate cells silence the spindle checkpoint, although it is not essential for silencing [30]. However, there is no evidence that dynein is involved in checkpoint protein targeting in yeast mitosis [31]. We tested dynein, *klp2*, *klp5*, and *klp6* mutants and found no effect on Mad2 localization in our synthetic checkpoint strain (see Figure S3G). An interaction between Mad1 and Cut7 (fission yeast Kinesin 5) was recently reported by Watanabe et al. [32]. They found that recruitment of Cut7 to kinetochores was Mad1 dependent, and that this interaction could be disrupted through mutation of the Mad1 N terminus (with the *mad1-KAKA* mutation) without affecting spindle checkpoint function. We note that the Cut7 kinesin motor has been demonstrated to be bi-directional in vitro [33] and that this motor localizes to spindle poles in addition to the spindle and midzone [34]. When we introduced the *mad1-KAKA* allele into our synthetic checkpoint system, we observed a dramatic decrease in spindle pole localization of Mad2-GFP (Figure 3B). Our interpretation is that fission yeast kinesin 5 is required for spindle pole enrichment of spindle checkpoint proteins in the synthetic arrest. However, imaging revealed that the *mad1-KAKA* cells still efficiently arrested at metaphase, with a diffuse nuclear pool of Mad2-RFP (Figures 3B and 3C). Thus, spindle pole enrichment of checkpoint proteins is not critical for the synthetic arrest, and we conclude that spindle poles are unlikely to be an important site of MCC generation in these cells. Mad1 and Mad2 interact with the nuclear periphery, via Mlp/TPR protein interactions [35, 36], and this has been demonstrated to be an important site of MCC assembly early in vertebrate mitosis [37]. Therefore, we analyzed another *mad1* mutant where the first 136 amino acids of Mad1 containing a coiled-coil region (CC) were removed, preventing Mad1-Mad2 interaction with Mlps and the nuclear envelope and also removing the Cut7 interaction site. These *mad1*-ΔCC cells were also able to arrest efficiently when TetR-Spc7-9TE and TetR-Δ(1-302)Mph1 were co-expressed (Figure 3E). We conclude that the Mad and Bub proteins do not need to be enriched at kinetochores, spindle poles, or the nuclear periphery for a robust checkpoint arrest to be generated in fission yeast. Most likely a diffuse, soluble pool of Spc7-Bub-Mad signaling assemblies is sufficient.

Checkpoint Signaling Generates a Mad1-Bub1 Complex and Is Inhibited by Bub3

A biochemical hallmark of active spindle checkpoint signaling in budding yeast is formation of a Bub1-Mad1 complex [20, 38], but this complex has proved challenging to detect in other systems. We immunoprecipitated Bub1-GFP from synthetically arrested cells (both with and without a tetO array), after cross-linking with dithiobis[succinimidylpropionate] (DSP) and were able to pull down complexes containing Mad1 and Mad2 (Figure 4A; data not shown). While our previous experiments suggested that this complex is rather labile in fission yeast extracts, we have also been able to co-immunoprecipitate these proteins in extracts made from *nda3* arrested cells after DSP cross-linking (data not shown). We propose that the synthetic checkpoint arrest is generated from a TetR-Spc7-Bub1 platform and that co-tethered TetR-Mph1 kinase then activates this further by phosphorylating Bub1 [20] to recruit the Mad1-Mad2 complex (Figure 4F). To directly test the importance of the Bub1-Mad1 interaction, we used the *bub1-CD1* mutant, where conserved phospho-sites thought to be needed for Mad1 interaction have been mutated to alanine [20, 39], and we found that these cells were unable to checkpoint arrest (Figure 4B). Co-immunoprecipitation experiments confirm that the Mad1-Bub1 interaction is efficiently generated from the TetR-Spc7-wt platform (data not shown), consistent with our ability to detect Mad2-GFP on the tetO array in the cells with tethered TetR-Spc7-wt (see Figure S2E). Detailed structural studies will be needed to explain this intriguing, partial "separation of function" with the Spc7-9TE allele: it recruits Bub1 better than Spc7-wt to the tetO array (Figure 1), yet it is less effective at recruiting Mad1&2 than Spc7-wt.

Watanabe et al. proposed that Bub3 might act as a chaperone to "suppress the ectopic activation of non-kinetochore Bub1" [6]. If so, one would expect to see a significant effect on the efficiency of ectopic TetR-Spc7-TetR-Mph1-induced checkpoint arrest in *bub3*Δ cells. Consistent with this prediction, Figure 4C demonstrates a striking advance (by ~4 hr) in the timing of arrest in *bub3*Δ cells arresting due to Spc7-9TE cells (although there is no effect with Spc7-wt, see Figure S4C). Deletion of *bub3* even allowed TetR-Spc7-9TA, TetR-Δ(1-302)Mph1 to arrest cells, again demonstrating the inhibitory effect of Bub3 (Figure 4D). Figure 4E shows a corresponding increase in the level of the Mad1-Bub1 complex in *bub3*Δ cells. We also note that in *bub3*Δ cells Bub1-GFP becomes hyperphosphorylated during mitotic arrest. This suggests one possible mode of Bub3 action: Bub3 binding might inhibit Bub1 auto-phosphorylation and thereby negatively impact Mad1p binding (see model in Figure 4F). We conclude that Bub3 likely acts to prevent ectopic spindle checkpoint signaling. Future experiments will address whether it does this by inhibiting the checkpoint activation pathway, or enhancing spindle checkpoint silencing [24]. In normal cells, Bub3 would prevent early nucleoplasmic signaling, and this effect would later be overcome when Mad-Bub complexes assemble at kinetochores and Spc7-Bub3-Bub1 interactions induce conformational changes in the Bub proteins, thereby activating Bub1 for downstream signaling. These Bub3 findings from our synthetic arrest are entirely consistent with a recent study published while our manuscript was in revision [40].

Conclusions

We have assembled a simple, synthetic, signaling system (SynCheck), avoiding the complexities of kinetochores, and generated a robust checkpoint arrest in fission yeast cells. $KNL1^{Spc7}$ acts as a platform to recruit Bub complexes and co-targeted $Mps1^{Mph1}$ kinase is sufficient to activate them for downstream signaling. This leads to assembly of a Mad1-Bub1 complex, MCC generation, and metaphase arrest. We note that the resulting cells arrest for several hours and eventually *cut* and die. This is possibly due to inefficient checkpoint silencing and that is currently under investigation.

It is clear from this and previous studies that checkpoint signals can be initiated from several sites: kinetochores, nuclear pores, possibly spindle poles, a tetO array, and soluble heterodimers of $KNL1^{Spc7}$-$Mps1^{Mph1}$ in the nucleoplasm. For a field

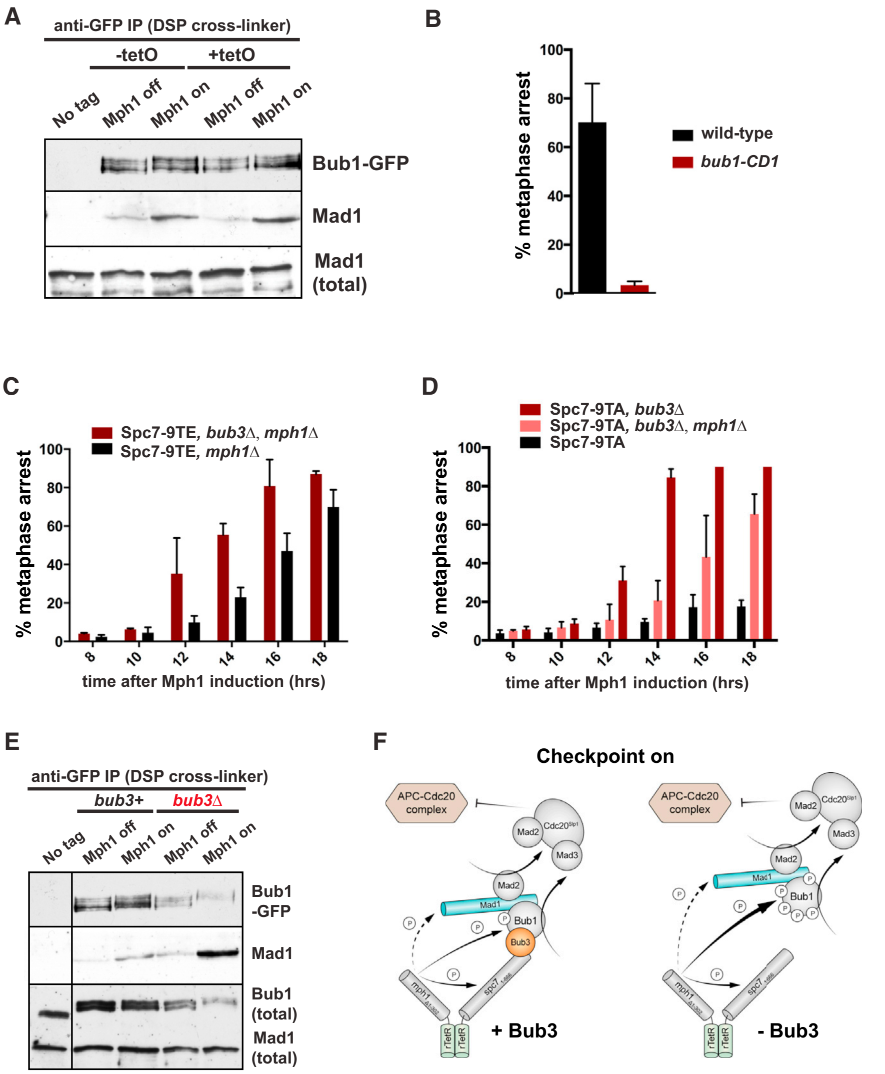

Figure 4. Synthetic Checkpoint Arrest Requires Mad1-Bub1 Complex Formation and Is Inhibited by Bub3

(A) Cells containing Bub1-GFP, TetR-Spc7-9TE, and TetR-Mph1ΔN were arrested as above for 16 hr then cross-linked with DSP before harvesting. Mad1 co-immunoprecipitates with Bub1-GFP in the arrested cells, both in the presence and absence of the tetO array. On the immunoblots, Bub1-GFP was detected with anti-Bub1 antibody and Mad1 with anti-Mad1 (see Supplemental Experimental Procedures).

(B) There is no arrest in the *bub1-CD1* mutant, which disrupts the Bub1-Mad1 interaction (see Figure S4A for images). This experiment was repeated three times, and data were plotted as the mean ± SD. The mutant Bub1-CD1 protein is stable (see Figure S4B).

(C) *bub3Δ* mutants containing TetR-Spc7-9TE arrest significantly faster than *bub3*$^{+}$, TetR-Spc7-9TE. This experiment was repeated three times, and data were plotted as the mean ± SD.

(D) TetR-Spc7-T9A combined with TetR-Mph1ΔN is able to arrest cells in the absence of Bub3. This experiment was repeated three times, and data were plotted as the mean ± SD.

(E) Higher levels of the Mad1-Bub1 complex are generated in *bub3Δ* cells. These cells contained TetR-Mph1ΔN and TetR-Spc7-9TE and were harvested after 12 hr of TetR-Mph1 induction. On the immunoblots, Bub1-GFP was detected with polyclonal anti-Bub1 antibodies, and Mad1 with anti-Mad1 antibodies (see Supplemental Experimental Procedures).

(F) Working model: diffusible heterodimers of TetR-Mph1ΔN and TetR-Spc7(1-666) actively produce a phospho-dependent Bub1-Mad1 complex, that than acts as an assembly platform for MCC production. In the absence of Bub3, shown to the right, Bub1 becomes hyper-phosphorylated, which can enhance Bub1-Mad1 complex assembly and MCC production.

See also Figure S4.

that often equates kinetochore localization with checkpoint action, it is rather humbling to observe that none of this localized enrichment is necessary for checkpoint arrest, at least in the relatively small yeast cells studied here. It will be very interesting to see whether similar ectopic platforms can arrest larger vertebrate cells and, if so, whether apoptosis is induced as this could have therapeutic implications.

SUPPLEMENTAL INFORMATION

Supplemental Information includes four figures and Supplemental Experimental Procedures and can be found with this article online at http://dx.doi.org/10.1016/j.cub.2016.11.014.

AUTHOR CONTRIBUTIONS

This project was conceived by K.G.H. Most of the experiments were carried out by I.L. and I.Y., with the exception of *mad1ΔCC* and some *bub3Δ* experiments (K.M.M.), Bub1-Mad1 co-immunoprecipitations (P.A.), some *bub3Δ*, *sgo2Δ* and Cen2-GFP experiments (S.S.N.C. and I.L.), and the kinesin and dynein motor mutant analyses (E.Z. and I.L.). The manuscript was written by K.G.H. with help from I.Y., I.L., K.M.M., P.A., and S.S.N.C.

ACKNOWLEDGMENTS

We thank Robin Allshire, Yoshi Watanabe, and Silke Hauf for yeast strains; Robin Allshire, Ken Sawin, and Jonathan Millar for plasmids; Dave Kelly for imaging advice; Rachael Barton for cross-linking advice; and all members of K.G.H.'s group for their encouragement and comments on the manuscript. This work was supported by a Seed Award from the Wellcome Trust to K.G.H. (108105) and the Wellcome Trust Centre for Cell Biology core grant (092076). I.L. is supported by the Darwin Trust of Edinburgh, P.A. by the Medical Research Council (MR/K501293/1), and S.S.N.C. by the Wellcome Trust (105258).

Received: April 26, 2016
Revised: October 14, 2016
Accepted: November 3, 2016
Published: December 22, 2016

REFERENCES

1. Jia, L., Kim, S., and Yu, H. (2013). Tracking spindle checkpoint signals from kinetochores to APC/C. Trends Biochem. Sci. *38*, 302–311.
2. London, N., and Biggins, S. (2014). Signalling dynamics in the spindle checkpoint response. Nat. Rev. Mol. Cell Biol. *15*, 736–747.

3. Musacchio, A. (2015). The molecular biology of spindle assembly checkpoint signaling dynamics. Curr. Biol. *25*, R1002–R1018.
4. Shepperd, L.A., Meadows, J.C., Sochaj, A.M., Lancaster, T.C., Zou, J., Buttrick, G.J., Rappsilber, J., Hardwick, K.G., and Millar, J.B. (2012). Phosphodependent recruitment of Bub1 and Bub3 to Spc7/KNL1 by Mph1 kinase maintains the spindle checkpoint. Curr. Biol. *22*, 891–899.
5. London, N., Ceto, S., Ranish, J.A., and Biggins, S. (2012). Phosphoregulation of Spc105 by Mps1 and PP1 regulates Bub1 localization to kinetochores. Curr. Biol. *22*, 900–906.
6. Yamagishi, Y., Yang, C.H., Tanno, Y., and Watanabe, Y. (2012). MPS1/Mph1 phosphorylates the kinetochore protein KNL1/Spc7 to recruit SAC components. Nat. Cell Biol. *14*, 746–752.
7. Vleugel, M., Omerzu, M., Groenewold, V., Hadders, M.A., Lens, S.M., and Kops, G.J. (2015). Sequential multisite phospho-regulation of KNL1-BUB3 interfaces at mitotic kinetochores. Mol. Cell *57*, 824–835.
8. Shah, J.V., Botvinick, E., Bonday, Z., Furnari, F., Berns, M., and Cleveland, D.W. (2004). Dynamics of centromere and kinetochore proteins; implications for checkpoint signaling and silencing. Curr. Biol. *14*, 942–952.
9. Howell, B.J., Moree, B., Farrar, E.M., Stewart, S., Fang, G., and Salmon, E.D. (2004). Spindle checkpoint protein dynamics at kinetochores in living cells. Curr. Biol. *14*, 953–964.
10. De Antoni, A., Pearson, C.G., Cimini, D., Canman, J.C., Sala, V., Nezi, L., Mapelli, M., Sironi, L., Faretta, M., Salmon, E.D., and Musacchio, A. (2005). The Mad1/Mad2 complex as a template for Mad2 activation in the spindle assembly checkpoint. Curr. Biol. *15*, 214–225.
11. Mapelli, M., Massimiliano, L., Santaguida, S., and Musacchio, A. (2007). The Mad2 conformational dimer: Structure and implications for the spindle assembly checkpoint. Cell *131*, 730–743.
12. Yang, M., Li, B., Liu, C.J., Tomchick, D.R., Machius, M., Rizo, J., Yu, H., and Luo, X. (2008). Insights into mad2 regulation in the spindle checkpoint revealed by the crystal structure of the symmetric mad2 dimer. PLoS Biol. *6*, e50.
13. Sudakin, V., Chan, G.K., and Yen, T.J. (2001). Checkpoint inhibition of the APC/C in HeLa cells is mediated by a complex of BUBR1, BUB3, CDC20, and MAD2. J. Cell Biol. *154*, 925–936.
14. Hardwick, K.G., Johnston, R.C., Smith, D.L., and Murray, A.W. (2000). MAD3 encodes a novel component of the spindle checkpoint which interacts with Bub3p, Cdc20p, and Mad2p. J. Cell Biol. *148*, 871–882.
15. Chao, W.C., Kulkarni, K., Zhang, Z., Kong, E.H., and Barford, D. (2012). Structure of the mitotic checkpoint complex. Nature *484*, 208–213.
16. Samejima, I., Spanos, C., Alves, Fde.L., Hori, T., Perpelescu, M., Zou, J., Rappsilber, J., Fukagawa, T., and Earnshaw, W.C. (2015). Whole-proteome genetic analysis of dependencies in assembly of a vertebrate kinetochore. J. Cell Biol. *211*, 1141–1156.
17. Biggins, S. (2013). The composition, functions, and regulation of the budding yeast kinetochore. Genetics *194*, 817–846.
18. Ito, D., Saito, Y., and Matsumoto, T. (2012). Centromere-tethered Mps1 pombe homolog (Mph1) kinase is a sufficient marker for recruitment of the spindle checkpoint protein Bub1, but not Mad1. Proc. Natl. Acad. Sci. USA *109*, 209–214.
19. Maldonado, M., and Kapoor, T.M. (2011). Constitutive Mad1 targeting to kinetochores uncouples checkpoint signalling from chromosome biorientation. Nat. Cell Biol. *13*, 475–482.
20. London, N., and Biggins, S. (2014). Mad1 kinetochore recruitment by Mps1-mediated phosphorylation of Bub1 signals the spindle checkpoint. Genes Dev. *28*, 140–152.
21. Heinrich, S., Windecker, H., Hustedt, N., and Hauf, S. (2012). Mph1 kinetochore localization is crucial and upstream in the hierarchy of spindle assembly checkpoint protein recruitment to kinetochores. J. Cell Sci. *125*, 4720–4727.
22. Joglekar, A.P., Bouck, D., Finley, K., Liu, X., Wan, Y., Berman, J., He, X., Salmon, E.D., and Bloom, K.S. (2008). Molecular architecture of the kinetochore-microtubule attachment site is conserved between point and regional centromeres. J. Cell Biol. *181*, 587–594.
23. Tange, Y., and Niwa, O. (2008). *Schizosaccharomyces pombe* Bub3 is dispensable for mitotic arrest following perturbed spindle formation. Genetics *179*, 785–792.
24. Vanoosthuyse, V., Meadows, J.C., van der Sar, S.J., Millar, J.B., and Hardwick, K.G. (2009). Bub3p facilitates spindle checkpoint silencing in fission yeast. Mol. Biol. Cell *20*, 5096–5105.
25. Aravamudhan, P., Goldfarb, A.A., and Joglekar, A.P. (2015). The kinetochore encodes a mechanical switch to disrupt spindle assembly checkpoint signalling. Nat. Cell Biol. *17*, 868–879.
26. Liu, X., McLeod, I., Anderson, S., Yates, J.R., 3rd, and He, X. (2005). Molecular analysis of kinetochore architecture in fission yeast. EMBO J. *24*, 2919–2930.
27. Flory, M.R., Morphew, M., Joseph, J.D., Means, A.R., and Davis, T.N. (2002). Pcp1p, an Spc110p-related calmodulin target at the centrosome of the fission yeast Schizosaccharomyces pombe. Cell Growth Differ. *13*, 47–58.
28. Mayer, C., Filopei, J., Batac, J., Alford, L., and Paluh, J.L. (2006). An extended anaphase signaling pathway for Mad2p includes microtubule organizing center proteins and multiple motor-dependent transitions. Cell Cycle *5*, 1456–1463.
29. Howell, B.J., McEwen, B.F., Canman, J.C., Hoffman, D.B., Farrar, E.M., Rieder, C.L., and Salmon, E.D. (2001). Cytoplasmic dynein/dynactin drives kinetochore protein transport to the spindle poles and has a role in mitotic spindle checkpoint inactivation. J. Cell Biol. *155*, 1159–1172.
30. Vanoosthuyse, V., and Hardwick, K.G. (2009). Overcoming inhibition in the spindle checkpoint. Genes Dev. *23*, 2799–2805.
31. Courtheoux, T., Gay, G., Reyes, C., Goldstone, S., Gachet, Y., and Tournier, S. (2007). Dynein participates in chromosome segregation in fission yeast. Biol. Cell *99*, 627–637.
32. Akera, T., Goto, Y., Sato, M., Yamamoto, M., and Watanabe, Y. (2015). Mad1 promotes chromosome congression by anchoring a kinesin motor to the kinetochore. Nat. Cell Biol. *17*, 1124–1133.
33. Edamatsu, M. (2014). Bidirectional motility of the fission yeast kinesin-5, Cut7. Biochem. Biophys. Res. Commun. *446*, 231–234.
34. Hagan, I., and Yanagida, M. (1990). Novel potential mitotic motor protein encoded by the fission yeast cut7+ gene. Nature *347*, 563–566.
35. Iouk, T., Kerscher, O., Scott, R.J., Basrai, M.A., and Wozniak, R.W. (2002). The yeast nuclear pore complex functionally interacts with components of the spindle assembly checkpoint. J. Cell Biol. *159*, 807–819.
36. Lee, S.H., Sterling, H., Burlingame, A., and McCormick, F. (2008). Tpr directly binds to Mad1 and Mad2 and is important for the Mad1-Mad2-mediated mitotic spindle checkpoint. Genes Dev. *22*, 2926–2931.
37. Rodriguez-Bravo, V., Maciejowski, J., Corona, J., Buch, H.K., Collin, P., Kanemaki, M.T., Shah, J.V., and Jallepalli, P.V. (2014). Nuclear pores protect genome integrity by assembling a premitotic and Mad1-dependent anaphase inhibitor. Cell *156*, 1017–1031.
38. Brady, D.M., and Hardwick, K.G. (2000). Complex formation between Mad1p, Bub1p and Bub3p is crucial for spindle checkpoint function. Curr. Biol. *10*, 675–678.
39. Heinrich, S., Sewart, K., Windecker, H., Langegger, M., Schmidt, N., Hustedt, N., and Hauf, S. (2014). Mad1 contribution to spindle assembly checkpoint signalling goes beyond presenting Mad2 at kinetochores. EMBO Rep. *15*, 291–298.
40. Mora-Santos, M.D., Hervas-Aguilar, A., Sewart, K., Lancaster, T.C., Meadows, J.C., and Millar, J.B. (2016). Bub3-Bub1 binding to Spc7/KNL1 toggles the spindle checkpoint switch by licensing the interaction of Bub1 with Mad1-Mad2. Curr. Biol. *26*, 2642–2650.

Current Biology, Volume *27*

Supplemental Information

Generation of a Spindle Checkpoint Arrest from Synthetic Signaling Assemblies

Ivan Yuan, Ioanna Leontiou, Priya Amin, Karen M. May, Sadhbh Soper Ní Chafraidh, Eliška Zlámalová, and Kevin G. Hardwick

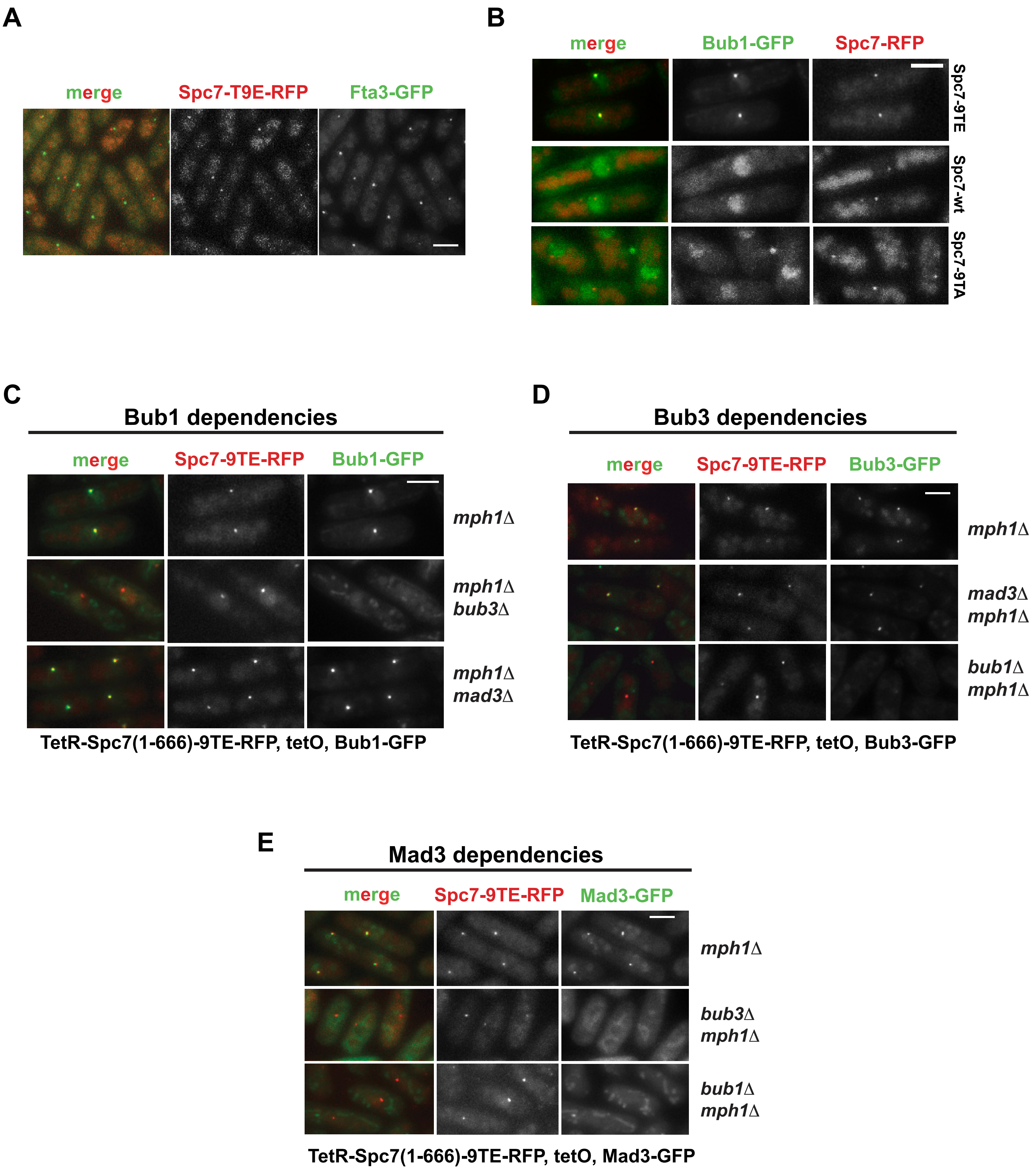
A
merge
Spc7-T9E-RFP
Fta3-GFP
B
merge
Bub1-GFP
Spc7-RFP
Spc7-9TE
Spc7-wt
Spc7-9TA
C
Bub1 dependencies
merge
Spc7-9TE-RFP
Bub1-GFP
mph1Δ
mph1Δ bub3Δ
mph1Δ mad3Δ
TetR-Spc7(1-666)-9TE-RFP, tetO, Bub1-GFP
D
Bub3 dependencies
merge
Spc7-9TE-RFP
Bub3-GFP
mph1Δ
mad3Δ mph1Δ
bub1Δ mph1Δ
TetR-Spc7(1-666)-9TE-RFP, tetO, Bub3-GFP
E
Mad3 dependencies
merge
Spc7-9TE-RFP
Mad3-GFP
mph1Δ
bub3Δ mph1Δ
bub1Δ mph1Δ
TetR-Spc7(1-666)-9TE-RFP, tetO, Mad3-GFP

Figure S1

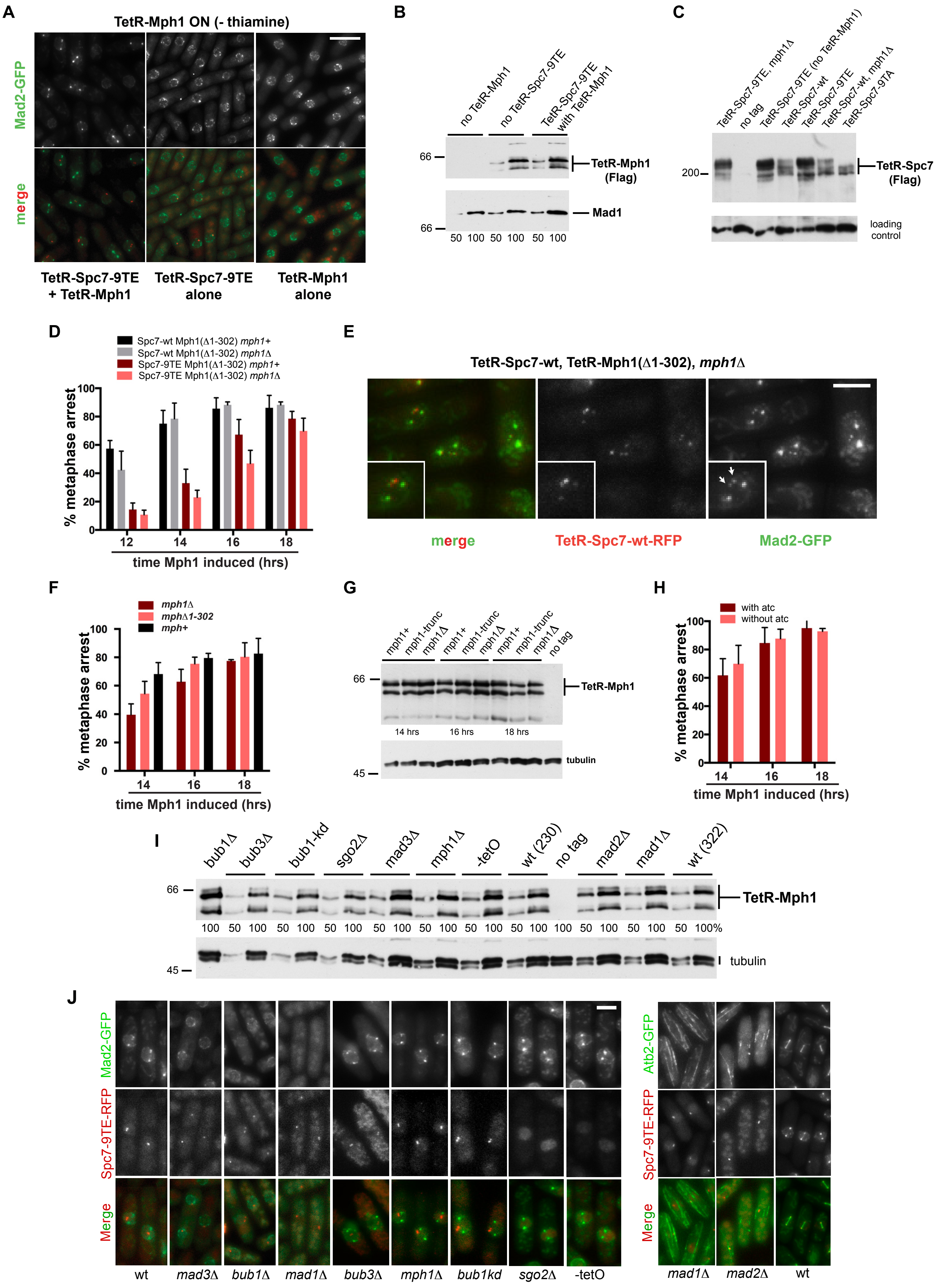
A
TetR-Mph1 ON (- thiamine)
Mad2-GFP
merge
TetR-Spc7-9TE + TetR-Mph1
TetR-Spc7-9TE alone
TetR-Mph1 alone
B
no TetR-Mph1
no TetR-Spc7-9TE
TetR-Spc7-9TE with TetR-Mph1
66
TetR-Mph1 (Flag)
Mad1
50 100 50 100 50 100
C
TetR-Spc7-9TE, mph1Δ
no tag
TetR-Spc7-9TE (no TetR-Mph1)
TetR-Spc7-wt
TetR-Spc7-9TE
TetR-Spc7-wt, mph1Δ
TetR-Spc7-9TA
200
TetR-Spc7 (Flag)
loading control
D
Spc7-wt Mph1(Δ1-302) mph1+
Spc7-wt Mph1(Δ1-302) mph1Δ
Spc7-9TE Mph1(Δ1-302) mph1+
Spc7-9TE Mph1(Δ1-302) mph1Δ
% metaphase arrest
time Mph1 induced (hrs)
E
TetR-Spc7-wt, TetR-Mph1(Δ1-302), mph1Δ
merge
TetR-Spc7-wt-RFP
Mad2-GFP
F
mph1Δ
mphΔ1-302
mph+
% metaphase arrest
time Mph1 induced (hrs)
G
mph1+ mph1-trunc mph1Δ
TetR-Mph1
14 hrs 16 hrs 18 hrs
no tag
tubulin
66
45
H
with atc
without atc
% metaphase arrest
time Mph1 induced (hrs)
I
bub1Δ bub3Δ bub1-kd sgo2Δ mad3Δ mph1Δ -tetO wt (230) no tag mad2Δ mad1Δ wt (322)
TetR-Mph1
100 50 100 50 100 50 100 50 100 50 100 50 100 50 100 100 50 100 50 100 50 100%
tubulin
66
45
J
Mad2-GFP
Spc7-9TE-RFP
Merge
wt mad3Δ bub1Δ mad1Δ bub3Δ mph1Δ bub1kd sgo2Δ -tetO
Atb2-GFP
Spc7-9TE-RFP
Merge
mad1Δ mad2Δ wt

Figure S2

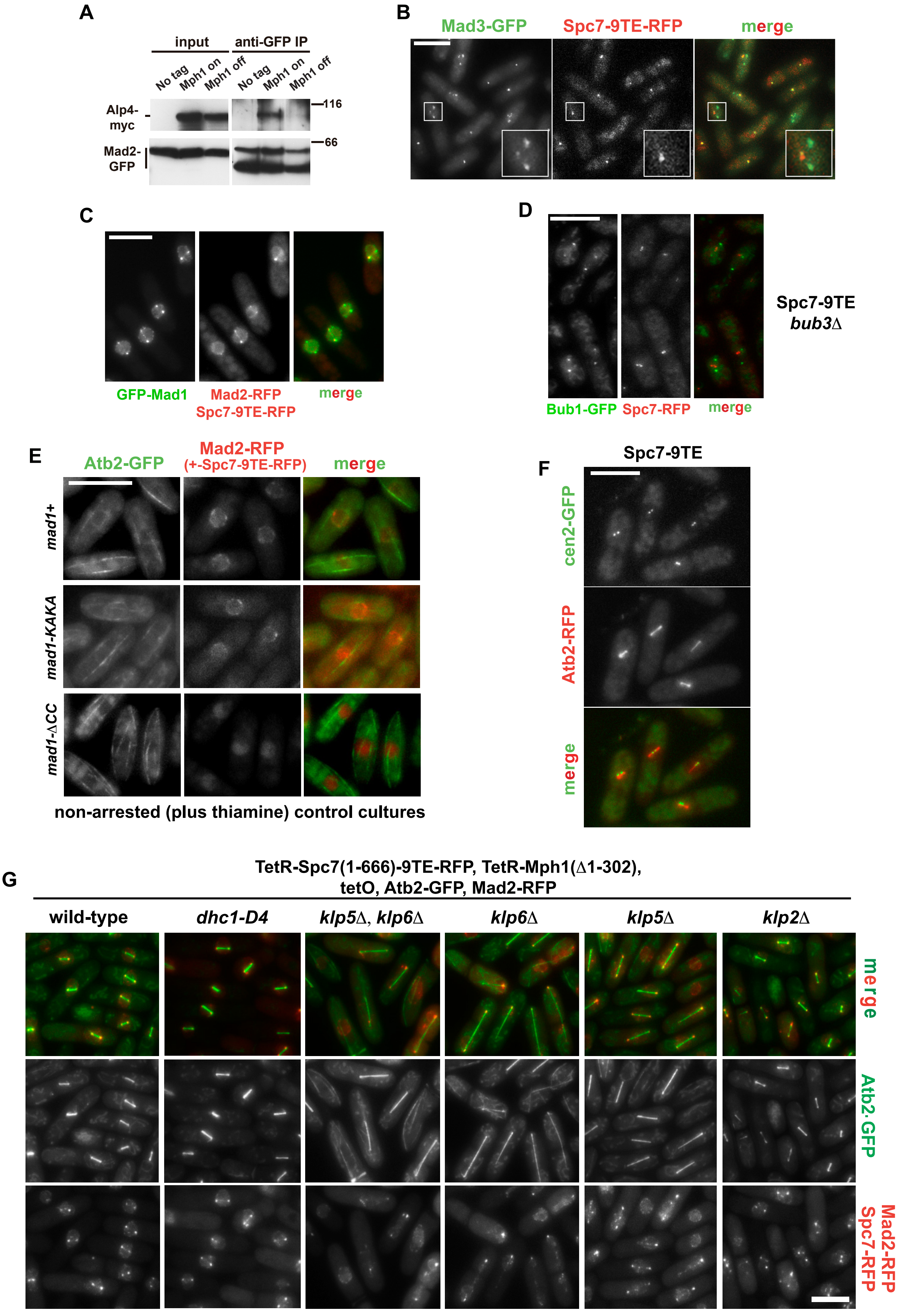
A
input
anti-GFP IP
No tag
Mph1 on
Mph1 off
No tag
Mph1 on
Mph1 off
Alp4-myc
Mad2-GFP
116
66
B
Mad3-GFP
Spc7-9TE-RFP
merge
C
GFP-Mad1
Mad2-RFP
Spc7-9TE-RFP
merge
D
Spc7-9TE
bub3Δ
Bub1-GFP
Spc7-RFP
merge
E
Atb2-GFP
Mad2-RFP
(+-Spc7-9TE-RFP)
merge
mad1+
mad1-KAKA
mad1-ΔCC
non-arrested (plus thiamine) control cultures
F
Spc7-9TE
cen2-GFP
Atb2-RFP
merge
G
TetR-Spc7(1-666)-9TE-RFP, TetR-Mph1(Δ1-302),
tetO, Atb2-GFP, Mad2-RFP
wild-type
dhc1-D4
klp5Δ, klp6Δ
klp6Δ
klp5Δ
klp2Δ
merge
Atb2-GFP
Mad2-RFP
Spc7-RFP

Figure S3

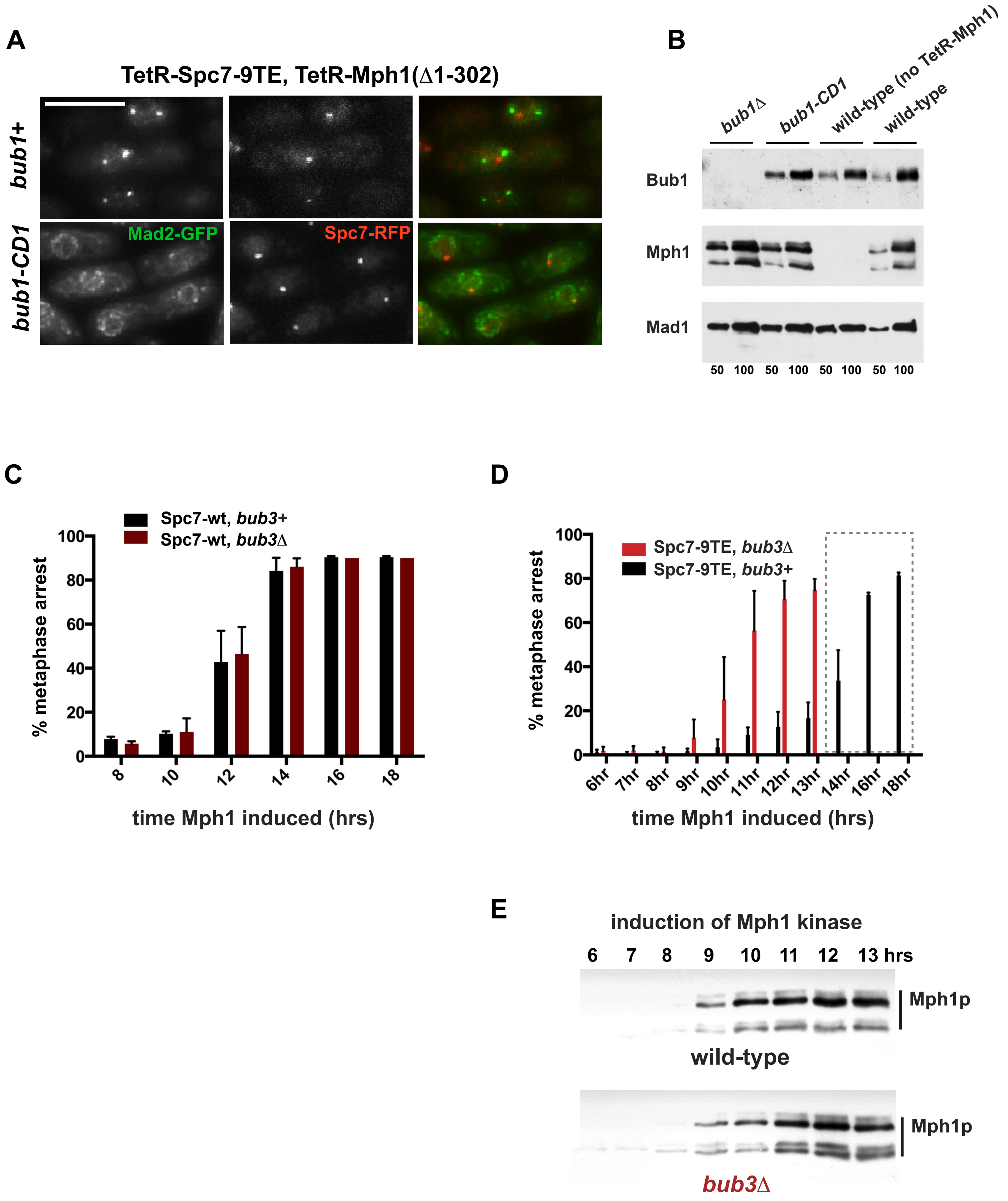
A
TetR-Spc7-9TE, TetR-Mph1(Δ1-302)
bub1+
bub1-CD1
Mad2-GFP
Spc7-RFP
B
bub1Δ
bub1-CD1
wild-type (no TetR-Mph1)
wild-type
Bub1
Mph1
Mad1
50 100 50 100 50 100 50 100
C
Spc7-wt, bub3+
Spc7-wt, bub3Δ
% metaphase arrest
time Mph1 induced (hrs)
D
Spc7-9TE, bub3Δ
Spc7-9TE, bub3+
% metaphase arrest
6hr 7hr 8hr 9hr 10hr 11hr 12hr 13hr 14hr 16hr 18hr
time Mph1 induced (hrs)
E
induction of Mph1 kinase
6 7 8 9 10 11 12 13 hrs
Mph1p
wild-type
Mph1p
bub3Δ

Figure S4

Supplemental Figure Legends

Figure S1, related to Figure 1
A) Tethered Spc7-RFP does not co-localise with the kinetochore marker Fta3-GFP.
Scale bars are 5 microns.
B) Comparison of the ability of TetR-Spc7-9TE, TetR-Spc7-wt and TetR-Spc7-9TA to recruit Bub1-GFP to the tetO array. NB. These strains all lack endogenous *mph1*.
C) Bub1-GFP dependencies: Bub1-GFP recruitment to Spc7-9TE is dependent on Bub3 but not Mad3 or Mph1.
D) Bub3-GFP dependencies: Bub3-GFP recruitment to Spc7-9TE is independent of endogenous Mph1 kinase and Mad3, but is dependent on Bub1.
E) Mad3-GFP dependencies: Mad3-GFP recruitment to Spc7-9TE is independent of endogenous Mph1 kinase, but is dependent on both Bub1 and Bub3.

Figure S2, related to Figure 2
A) Mad2-GFP was observed at spindle poles in cells that have arrested due to co-tethering of TetR-Spc7-9TE and TetR-Mph1. This was not observed in the absence of either of the Mph1 or Spc7 fusion proteins. Scale bar is 10 microns.
B) Immunoblots (anti-Flag) demonstrating similar expression of TetR-Mph1(Δ1-302) in different strains. 50 and 100 refer to 50% and 100% equivalent loading.
C) Immunoblot (anti-Flag) demonstrating the expression levels and relative gel-shifts of TetR-Spc7 constructs. TetR-Spc7-9TE has lower gel-mobility, and TetR-Spc7-9TA faster gel-mobility.
D) Comparison of the ability of TetR-Spc7-9TE and TetR-Spc7-wt to arrest cells, both with and without an endogenous copy of the *mph1+* gene. The wild-type Spc7 arrests faster than Spc7-9TE. This experiment was repeated 8 times and data plotted as mean +/- SD.
E) Imaging demonstrates that Mad2-GFP does localise to the tetO array in strains expressing TetR-Spc7-wt-RFP, TetR-Δ(1-302)Mph1. White arrows indicate Mad2-GFP enriched on the tetO array. Interestingly, this can not be detected in Spc7-9TE arrests. Scale bar is 5 microns.
F) To further analyse the impact of differing Mph1 kinase levels on signalling output, we quantitated the TetR-Spc7-9TE, TetR-Δ(1-302)Mph1 arrest in three different genetic backgrounds: with endogenous Mph1 kinase deleted; with endogenous Mph1 N-terminally truncated (diffusible); and with an endogenous wild-type gene present (likely to be kinetochore localised). Increasing the level of endogenous Mph1 kinase in this way slightly advances the time at which cells arrest. This experiment was repeated 3 times and data plotted as mean +/- SD.
G) Immunoblots (anti-Flag) demonstrating equal expression levels of the tetR-Mph1(D1-302) fusion protein in strains with different *mph1* alleles at the endogenous *Mph1+* locus.
H) Comparison of the ability of tetR-Spc7-9TE and tetR-Spc7-wt to arrest cells, with and without the presence of anhydro-tetracycline (atc) in the growth media. This experiment was repeated 10 times and data plotted as mean +/- SD. No significant effect was observed (unpaired t-test).
I) Immunoblot (anti-Flag) demonstrating equal expression levels of the tetR-Mph1(Δ1-302) fusion protein in the *mad/bub/sgo* mutant strains. All strains contain the tetO array, apart from the far right control. All strains are expressing tetR-Spc7-T9E and tetR-Mph1(Δ1-302) fusion proteins. 50 and 100 represent 50 and 100% loading of samples.
J) No arrest was observed in *mad1*Δ, *mad2*Δ, *mad3*Δ, or *bub1*Δ strains co-expressing tetR-Spc7-9TE and tetR-Mph1(Δ1-302), but *mph1*Δ, *bub3*Δ, *bub1-kd, or sgo2*Δ strains did arrest efficiently. Arrest was scored using either Mad2-GFP (left panels), or Atb2-GFP (three right hand panels). All of these strains contain the tetO array, apart from the control strain labelled -tetO and *sgo2*Δ. Scale bar is 5 microns.

Figure S3, related to Figure 3
All the spindle checkpoint proteins accumulate at spindle poles.
A) Mad2-GFP was co-immunoprecipitated with the gamma tubulin complex component Alp4 in Spc7-Mph1 arrested-cells. Strains were arrested for 16 hours.
B) Mad3-GFP is seen at spindle poles and at the tetO array in TetR-Spc7-Mph1 arrested cells. Scale bar is 5 microns.
C) GFP-Mad1 (note that a C-terminal tag on Mad1 is not fully functional) accumulates at the spindle poles in arrested cells. Scale bar is 10 microns.

D) Bub1-GFP accumulates at spindle poles in a Bub3-independent fashion. This is consistent with the model whereby Mad1 moves to spindle poles in a Cut7-dependent manner, and takes bound Bub1 with it. Note, if present in cells, Bub3-GFP is also taken to the poles of arrested cells (not shown). Scale bar is 10 microns.
E) These strains have not had TetR-Mph1 induced (they are growing in media containing thiamine, and so don't arrest and display interphase microtubule arrays). Mad2-GFP decorates the nuclear periphery in the *mad1-KAKA* allele, but is found to be diffuse throughout the nucleus in the *mad1-ΔCC1* allele. This allele will be described in detail in another publication (KM and KGH, in preparation). Scale bar is 10 microns.
F) In these arrested strains (containing TetR-Spc7-9TE and TetR-Mph1(Δ1-302)), cen2-GFP [S1] images demonstrate that chromosome 2 is efficiently bi-oriented on the short metaphase spindle (Atb2-RFP). Thus the synthetic arrest isn't perturbing kinetochore microtubule interactions. Scale bar is 5 microns.
G) *klp2, klp5, klp6* and dynein motor mutations[S2] did not affect the movement of Mad2-RFP to spindle poles. Scale bar is 5 microns. Note that the metaphase spindle length is significantly affected in some of these kinesin mutants, as has been previously reported [S3-S5]. Scale bar is 10 microns.

Figure S4, related to Figure 4
A) **Cells containing the *bub1-CD1* allele are unable to arrest** when co-expressing TetR-Spc7-9TE and TetR-Mph1(Δ1-302), demonstrating that the Mad1-Bub1 interaction is necessary to arrest such cells, as it is for other checkpoint arrests [S6]. Scale bar is 10 microns.
B) Immunoblots demonstrating equivalent expression of Bub1, Mph1, and Mad1 in these strains. Bub1 and Mad1 were detected with our own polyclonal antibodies (these are new Bub1 antibodies, made as previously described [S7]), and Mph1 with anti-Flag.
C) The TetR-Spc7-wt, TetR-Mph1(Δ1-302) arrest is not advanced in the absence of Bub3p. This experiment was performed 3 times and is plotted as the mean +/- SD at each time point. Note, this is unlike the effects on Spc7-9TE and Spc7-9TA arrests which were significantly advanced (Figure 4). Presumably this means that the Spc7-wt function is not rate-limiting in checkpoint arresting cells.
D) The effect of Bub3, in advancing the Spc7-9TE arrest, is also observed in the presence of endogenous Mph1 kinase. This experiment was repeated seven times and is plotted as the mean +/- SD at each time point. The later time points (within the dashed box) were usually scored from cultures that had been induced earlier.
E) Immunoblot (anti-Flag) demonstrating that Mph1 is induced with similar kinetics in strains being analysed (all were analysed, just Spc7-9TE +/- *bub3* are show here, for simplicity).

Supplemental Experimental Procedures

Construction of TetR fusion constructs

P_{nmt81}-2xFLAG-rTetR-mph1$_{\Delta 1\text{-}302}$

rTetR was amplified out from *pAK2* (gift from Alexander Kagansky, Allshire lab), digested with NheI and AseI, and inserted into a pHFF81C vector (gift from Ken Sawin) digested with NheI and NdeI. *mph1*$_{\Delta 1\text{-}302}$ was amplified from genomic DNA (strain from Silke Hauf) and inserted into tTetR-pHFF81C using Gateway recombination.

pLYS1U-P_{adh15}-NLS-2xFLAG-rTetR-spc7$_{1\text{-}666}$

pRAD15 (gift from Robin Allshire) was amplified using phosphorylated primers before DpnI digestion and re-ligation to re-create the vector with NheI and XhoI sites. These sites were then used to insert a PCR fragment *NLS-rtTA-mCherry-FLAG-ccdB* into *pRAD15*. *P_{adh15}-NLS-rtTA-mCherry-2xFLAG-ccdB* was then amplified out and subsequently joined to a PCR fragment containing the *pLYS1U* backbone (gift from Jonathan Millar) using KpnI and XhoI to form *pLYS1U-P_{adh15}-NLS-2xFLAG-rTetR-ccdB*. *spc7$_{1\text{-}666}$*(wild-type, -9TE and -9TA) were then inserted into this vector by Gateway recombination.

Construction of the Mad1 N-terminal truncation (*mad1-ΔCC*)

To truncate Mad1 expressed from its endogenous promotor, 762bp containing the promotor region, 325bp of flanking sequence upstream of this and 566bp of *mad1* coding sequence, excluding the first 500bp of *mad1*, were amplified from genomic DNA. The hygromycin resistance cassette was amplified from pFA6hphMX. The flanking sequence was digested with Kpn1 and Sal1 and cloned into pBluescript, then the resulting vector was digested with Sal1 and EcoRV and the remaining fragments were assembled by Gibson Assembly (NEB) in the following order; hygR, promotor then coding region, to give pMad1Δ1cc-hyg. To GFP tag the N-terminus of full length Mad1 the endogenous promotor was replaced by *Padh21* and *gfp* sequences inserted before 1kb of *mad1^{WT}* coding sequence as described above, using Sal1 and EcoRV digested vector and Gibson assembly. The assembled sequences were amplified by PCR and transformed into fission yeast. Standard yeast procedures, media and transformation protocols were used throughout this study.

Microscopy

For the *synthetic arrest*: cells were first grown on YES (rich) plates at 32°C overnight. The following morning, cells were transferred to 10 ml of liquid PMG medium containing 15μM thiamine and were incubated at 30°C with shaking. After seven hours, cells were harvested by spinning at 6,000 RPM for 2.5 min and washed 3 times with fresh PMG (containing supplements). The cells were transferred to fresh PMG (without thiamine) containing 10μM anhydrodoxycycline (Sigma), and then incubated at 30°C for 14, 16 and 18hrs.

Depending on the experiment the next morning the cells were harvested by spinning at 6,000 RPM for 2.5 min. The cells were washed with 1ml of clear PMG (without glucose) and harvested by spinning at 6,000 RPM for 1 min. The supernatant was removed (a small volume of media was left, depending on the pellet size) and 6-10μl of cells was deposited on a glass slide and covered with a glass coverslip. Cells were imaged immediately using a 100x oil immersion lens and a Zeiss Axiovert 200M microscope (Carl Zeiss Ltd.), equipped with a CoolSnap CCD camera (Photometrics) and Slidebook 5.0 software (3i, Intelligent Imaging Innovations, Inc.). Typical acquisition settings: 300 ms exposure (FITC & CY3), 2x binning, Z-series over 3 mm range in 0.5 mm steps (7 planes).

Crosslinking

Cells were checked under the microscope, harvested and re-suspended in reaction buffer (20mM Hepes pH7.6, 100mM KOAc). 20mM DSP (in DMSO) was added to the cells and the cross-linking reaction was left to shake slowly for 30 minutes (or 2 hours at 4°C). To quench the reaction, cells were pelleted, re-suspended in 100mM Tris pH 7.5 and left to shake for 15 minutes. Cells were pelleted at 4°C and frozen in dry ice.

Co-immunoprecipitation

The pellet was re-suspended in lysis buffer (50mM Hepes pH7.6, 75mM KCl, 1mM $MgCl_2$, 1mM EGTA, 10% Glycerol, 0.1% Triton X-100, 1mM Na_3VO_4, 10 μg/mL CLAAPE (protease inhibitor mix containing chymostatin, leupeptin, aprotinin, antipain, pepstatin, E-64 dissolved in DMSO at a concentration of 10 mg/mL), 1mM Pefabloc, 0.01mM microcystin). Zirconia beads were added to the cells and they were broken by bead-beating twice for 20 seconds and kept on ice for 30 seconds in between. Lysed cells were centrifuged at 14000 rpm for 10 minutes at 4°C to remove cell debris.

The clear lysate was incubated with anti-GFP-coupled Dynabeads for 15 minutes at 4oC. The bead were washed four times with wash buffer (50mM Hepes pH7.6, 75mM KCl, 1mM $MgCl_2$, 1mM EGTA, 10% Glycerol) and stored at -80°C until further use. Proteins were eluted by adding sample buffer containing DTT and standing at room temperature for 15 minutes, following this, they were then run on an SDS-PAGE gel.

List of the strains used in this study

Figure 1

IY184 *lys1::P_{adh15}-rtetR-mCherry-$spc7_{1-666}$-9TE:ura4 tetO:kanR bub1-GFP mph1Δ::natR*
IY188 *lys1::P_{adh15}-rtetR-mCherry-$spc7_{1-666}$-9TE:ura4 tetO:kanR bub3-GFP mph1Δ::natR*
IY185 *lys1::P_{adh15}-rtetR-mCherry-$spc7_{1-666}$-9TE:ura4 tetO:kanR mad3-GFP mph1Δ::natR*
IL322 *lys1::P_{adh15}-rtetR-mCherry-$spc7_{1-666}$-9TE:ura4 tetO:kanR leu1$^+$:P_{nmt81}rtetR-mph1(D1-302) GFP-atb2:leu+ mad2-RFP:natR*

Figure 2

IY230 *lys1::P_{adh15}-rtetR-mCherry-$spc7_{1-666}$-9TE:ura4 tetO:kanR leu1$^+$:P_{nmt81}rtetR-mph1(D1-302) mad2-GFP:his3*
IY232 *tetO:kanR, P_{nmt81}rtetR-mph1(D1-302) mad2-GFP:his3*
IL386 *lys1::P_{adh15}-rtetR-mCherry-$spc7_{1-666}$-9TE:ura4 tetO:kanR mad2-GFP:his3*
IL787 *lys1::P_{adh15}-rtetR-mCherry-$spc7_{1-666}$:ura4 tetO:kanR leu1$^+$:P_{nmt81}rtetR-mph1(D1-302) mad2-GFP:his3*
IL673 *lys1::P_{adh15}-rtetR-mCherry-$spc7_{1-666}$-9TA:ura4 tetO:kanR leu1$^+$:P_{nmt81}rtetR-mph1(D1-302) mad2-GFP:his3*
KM1437 *lys1::P_{adh15}-rtetR-mCherry-$spc7_{1-666}$:ura4 leu1$^+$:P_{nmt81}rtetR-mph1(D1-302) mad2-GFP:his3*
IY222 *lys1::P_{adh15}-rtetR-mCherry-$spc7_{1-666}$-9TE:ura4 leu1$^+$:P_{nmt81}rtetR-mph1(D1-302) mad2-GFP:his3*
IL473 *lys1::P_{adh15}-rtetR-mCherry-$spc7_{1-666}$-9TE:ura4 tetO:kanR leu1$^+$:P_{nmt81}-mph1(D1-302) mad2-GFP:his3*
IL146 *lys1::P_{adh15}-rtetR-mCherry-$spc7_{1-666}$-9TE:ura4 tetO:kanR leu1$^+$:P_{nmt81}rtetR-mph1(D1-302) mad3Δ::Ura4+ mad2-GFP:his3*
IL 245 *lys1::P_{adh15}-rtetR-mCherry-$spc7_{1-666}$-9TE:ura4 tetO:kanR leu1$^+$:P_{nmt81}rtetR-mph1(D1-302) bub1Δ::natR mad2-GFP:his3*
IY240 *lys1::P_{adh15}-rtetR-mCherry-$spc7_{1-666}$-9TE:ura4 tetO:kanR leu1$^+$:P_{nmt81}rtetR-mph1(D1-302) bub3Δ::hygR mad2-GFP:his3*
IL133 *lys1::P_{adh15}-rtetR-mCherry-$spc7_{1-666}$-9TE:ura4 tetO:kanR leu1$^+$:P_{nmt81}rtetR-mph1(D1-302) mph1Δ::natR mad2-GFP:his3*
IL277 *lys1::P_{adh15}-rtetR-mCherry-$spc7_{1-666}$-9TE:ura4 tetO:kanR leu1$^+$:P_{nmt81}rtetR-mph1(D1-302) bub1-kd mad2-GFP:his3*
IL322 *lys1::P_{adh15}-rtetR-mCherry-$spc7_{1-666}$-9TE:ura4 tetO:kanR leu1$^+$:P_{nmt81}rtetR-mph1(D1-302) GFP-atb2:leu+ mad2-RFP:natR*
IL604 *lys1::P_{adh15}-rtetR-mCherry-$spc7_{1-666}$-9TE:ura4 tetO:kanR leu1$^+$:P_{nmt81}rtetR-mph1(D1-302) GFP-atb2:leu+ mad2Δ::ura4+*
IL609 *lys1::P_{adh15}-rtetR-mCherry-$spc7_{1-666}$-9TE:ura4 tetO:kanR leu1$^+$:P_{nmt81}rtetR-mph1(D1-302) GFP-atb2:leu+ mad1Δ::hygR mad2-RFP:natR*
SS18 *lys1::P_{adh15}-rtetR-mCherry-$spc7_{1-666}$-9TE:ura4 leu1$^+$:P_{nmt81}rtetR-mph1(D1-302) sgo2Δ::natR mad2-GFP:his3*

Figure 3

IY239 *lys1::P_{adh15}-rtetR-mCherry-$spc7_{1-666}$-9TE:ura4 tetO:kanR leu1$^+$:P_{nmt81}rtetR-mph1(D1-302) pcp1-mcherry::natR mad2-GFP:his3*
IY242 *lys1::P_{adh15}-rtetR-mCherry-$spc7_{1-666}$-9TE:ura4 tetO:kanR leu1$^+$:P_{nmt81}rtetR-mph1(D1-302) fta3-tTd:natR mad2-GFP:his3*
IL322 *lys1::P_{adh15}-rtetR-mCherry-$spc7_{1-666}$-9TE:ura4 tetO:kanR leu1$^+$:P_{nmt81}rtetR-mph1(D1-302) GFP-atb2:leu+ mad2-RFP:natR*
IL634 *lys1::P_{adh15}-rtetR-mCherry-$spc7_{1-666}$-9TE:ura4 tetO:kanR leu1$^+$:P_{nmt81}rtetR-mph1(D1-302) GFP-atb2:leu+ mad1-KAKA mad2-RFP:natR*

KM1415 lys1::P_{adh15}-rtetR-mCherry-spc7$_{1-666}$-9TE:ura4 tetO:kanR leu1^{+}:P_{nmt81}rtetR-mph1(D1-302) GFP-atb2:leu+ mad1-Δcc:hygR mad2-RFP:natR

Figure 4

IL250 lys1::P_{adh15}-rtetR-mCherry-spc7$_{1-666}$-9TE:ura4 tetO:kanR leu1^{+}:P_{nmt81}rtetR-mph1(D1-302) bub1-GFP:his3
IL615 lys1::P_{adh15}-rtetR-mCherry-spc7$_{1-666}$-9TE:ura4 leu1^{+}:P_{nmt81}rtetR-mph1(D1-302) bub1-GFP:his3
IL382 lys1::P_{adh15}-rtetR-mCherry-spc7$_{1-666}$-9TE:ura4 tetO:kanR leu1^{+}:P_{nmt81}rtetR-mph1(D1-302)
IL626 lys1::P_{adh15}-rtetR-mCherry-spc7$_{1-666}$-9TE:ura4 tetO:kanR leu1^{+}:P_{nmt81}rtetR-mph1(D1-302) bub1CD1 mad2-GFP:his3
IY230 lys1::P_{adh15}-rtetR-mCherry-spc7$_{1-666}$-9TE:ura4 tetO:kanR leu1^{+}:P_{nmt81}rtetR-mph1(D1-302) mad2-GFP:his3
IL266 lys1::P_{adh15}-rtetR-mCherry-spc7$_{1-666}$-9TE:ura4 tetO:kanR leu1^{+}:P_{nmt81}rtetR-mph1(D1-302) bub3Δ::hygR bub1-GFP:his3
IL133 lys1::P_{adh15}-rtetR-mCherry-spc7$_{1-666}$-9TE:ura4 tetO:kanR leu1^{+}:P_{nmt81}rtetR-mph1(D1-302) mph1Δ::natR mad2-GFP:his3
IL738 lys1::P_{adh15}-rtetR-mCherry-spc7$_{1-666}$-9TE:ura4 tetO:kanR leu1^{+}:P_{nmt81}rtetR-mph1(D1-302) bub3Δ::hygR mph1Δ::natR mad2-GFP:his3
IL708 lys1::P_{adh15}-rtetR-mCherry-spc7$_{1-666}$-9TA:ura4 tetO:kanR leu1^{+}:P_{nmt81}rtetR-mph1(D1-302) bub3Δ::hygR mad2-GFP:his3
IL711 lys1::P_{adh15}-rtetR-mCherry-spc7$_{1-666}$-9TA:ura4 tetO:kanR leu1^{+}:P_{nmt81}rtetR-mph1(D1-302) bub3Δ::hygR mph1Δ::natR mad2-GFP:his3
IL673 lys1::P_{adh15}-rtetR-mCherry-spc7$_{1-666}$-9TA:ura4 tetO:kanR leu1^{+}:P_{nmt81}rtetR-mph1(D1-302) mad2-GFP:his3

Supplemental Figures

Figure S1

IY170 lys1::P_{adh15}-rtetR-mCherry-spc7$_{1-666}$-9TE:ura4 tetO:kanR fta3-GFP:kanR
IY184 lys1::P_{adh15}-rtetR-mCherry-spc7$_{1-666}$-9TE:ura4 tetO:kanR bub1-GFP mph1Δ::natR
IL272 lys1::P_{adh15}-rtetR-mCherry-spc7$_{1-666}$-9TE:ura4 tetO:kanR bub1-GFP:his3+ mph1Δ::natR bub3Δ::hygR
IY257 lys1::P_{adh15}-rtetR-mCherry-spc7$_{1-666}$-9TE:ura4 tetO:kanR bub1-GFP:his3+ mph1Δ::natR mad3Δ::ura4+
IY185 lys1::P_{adh15}-rtetR-mCherry-spc7$_{1-666}$-9TE:ura4 tetO:kanR mad3-GFP:his3+ mph1Δ::natR
IL433 lys1::P_{adh15}-rtetR-mCherry-spc7$_{1-666}$-9TE:ura4 tetO:kanR mad3-GFP:his3+ bub3Δ::hygR mph1Δ::natR
IL432 lys1::P_{adh15}-rtetR-mCherry-spc7$_{1-666}$-9TE:ura4 tetO:kanR mad3-GFP:his3+ bub1Δ::ura4+ mph1Δ::natR
IY188 lys1::P_{adh15}-rtetR-mCherry-spc7$_{1-666}$-9TE:ura4 tetO:kanR bub3-GFP:his3+ mph1Δ::natR
IL342 lys1::P_{adh15}-rtetR-mCherry-spc7$_{1-666}$-9TE:ura4 tetO:kanR bub3-GFP:his3+ mad3Δ::ura4+ mph1Δ::natR
IL404 lys1::P_{adh15}-rtetR-mCherry-spc7$_{1-666}$-9TE:ura4 tetO:kanR bub3-GFP:his3+ bub1Δ::ura4+ mph1Δ::natR
IL743 lys1::P_{adh15}-rtetR-mCherry-spc7$_{1-666}$-9TA:ura4 tetO:kanR mph1Δ::natR bub1-GFP:his3+
IL755 lys1::P_{adh15}-rtetR-mCherry-spc7$_{1-666}$:ura4 tetO:kanR mph1Δ::natR bub1-GFP:his3+

Figure S2

IY230 lys1::P_{adh15}-rtetR-mCherry-spc7$_{1-666}$-9TE:ura4 tetO:kanR leu1^{+}:P_{nmt81}rtetR-mph1(D1-302) mad2-GFP:his3
IY232 tetO:kanR leu1^{+}:P_{nmt81}rtetR-mph1(D1-302) mad2-GFP:his3
IL386 lys1::P_{adh15}-rtetR-mCherry-spc7$_{1-666}$-9TE:ura4 tetO:kanR mad2-GFP:his3
IL133 lys1::P_{adh15}-rtetR-mCherry-spc7$_{1-666}$-9TE:ura4 tetO:kanR leu1^{+}:P_{nmt81}rtetR-mph1(D1-302) mph1Δ::natR mad2-GFP:his3
KM10 ade6-210 leu1-32 ura4-D18
IL787 lys1::P_{adh15}-rtetR-mCherry-spc7$_{1-666}$:ura4 tetO:kanR leu1^{+}:P_{nmt81}rtetR-mph1(D1-302) mad2-GFP:his3
IL673 lys1::P_{adh15}-rtetR-mCherry-spc7$_{1-666}$-9TA:ura4 tetO:kanR leu1^{+}:P_{nmt81}rtetR-mph1(D1-302) mad2-GFP:his3
IL788 lys1::P_{adh15}-rtetR-mCherry-spc7$_{1-666}$:ura4 tetO:kanR leu1^{+}:P_{nmt81}rtetR-mph1(D1-302) mph1Δ::natR mad2-GFP:his3
IY222 lys1::P_{adh15}-rtetR-mCherry-spc7$_{1-666}$-9TE:ura4 leu1^{+}:P_{nmt81}rtetR-mph1(D1-302) mad2-GFP:his3
IL322 lys1::P_{adh15}-rtetR-mCherry-spc7$_{1-666}$-9TE:ura4 tetO:kanR leu1^{+}:P_{nmt81}rtetR-mph1(D1-302) GFP-atb2:leu+ mad2-RFP:natR

IL619 *lys1::P_{adh15}-rtetR-mCherry-$spc7_{1-666}$-9TE:ura4 tetO:kanR $leu1^{+}$:P_{nmt81}rtetR-mph1(D1-302) mph1(D1-302)-GFP:G418, GFP-atb2:leu+ mad2-RFP:natR*

IL665 *lys1::P_{adh15}-rtetR-mCherry-$spc7_{1-666}$-9TE:ura4 tetO:kanR $leu1^{+}$:P_{nmt81}rtetR-mph1(D1-302) mph1Δ::natR, GFP-atb2:leu+ mad2-RFP:natR*

IL146 *lys1::P_{adh15}-rtetR-mCherry-$spc7_{1-666}$-9TE:ura4 tetO:kanR $leu1^{+}$:P_{nmt81}rtetR-mph1(D1-302) mad3Δ::ura4+ mad2-GFP:his3*

IL 245 *lys1::P_{adh15}-rtetR-mCherry-$spc7_{1-666}$-9TE:ura4 tetO:kanR $leu1^{+}$:P_{nmt81}rtetR-mph1(D1-302) bub1Δ::natR mad2-GFP:his3*

IY240 *lys1::P_{adh15}-rtetR-mCherry-$spc7_{1-666}$-9TE:ura4 tetO:kanR $leu1^{+}$:P_{nmt81}rtetR-mph1(D1-302) bub3Δ::hygR mad2-GFP:his3*

IL133 *lys1::P_{adh15}-rtetR-mCherry-$spc7_{1-666}$-9TE:ura4 tetO:kanR $leu1^{+}$:P_{nmt81}rtetR-mph1(D1-302) mph1Δ::natR mad2-GFP:his3*

IL277 *lys1::P_{adh15}-rtetR-mCherry-$spc7_{1-666}$-9TE:ura4 tetO:kanR $leu1^{+}$:P_{nmt81}rtetR-mph1(D1-302) bub1-kd mad2-GFP:his3*

IL322 *lys1::P_{adh15}-rtetR-mCherry-$spc7_{1-666}$-9TE:ura4 tetO:kanR $leu1^{+}$:P_{nmt81}rtetR-mph1(D1-302) GFP-atb2:leu+ mad2-RFP:natR*

IL604 *lys1::P_{adh15}-rtetR-mCherry-$spc7_{1-666}$-9TE:ura4 tetO:kanR $leu1^{+}$:P_{nmt81}rtetR-mph1(D1-302) GFP-atb2:leu+ mad2Δ::ura4+*

IL609 *lys1::P_{adh15}-rtetR-mCherry-$spc7_{1-666}$-9TE:ura4 tetO:kanR $leu1^{+}$:P_{nmt81}rtetR-mph1(D1-302) GFP-atb2:leu+ mad1Δ::hygR mad2-RFP:natR*

SS18 *lys1::P_{adh15}-rtetR-mCherry-$spc7_{1-666}$-9TE:ura4 $leu1^{+}$:P_{nmt81}rtetR-mph1(D1-302) sgo2Δ::natR mad2-GFP:his3*

Figure S3

IL511 *lys1::P_{adh15}-rtetR- mCherry-$spc7_{1-666}$-9TE:ura4 tetO:kanR $leu1^{+}$:P_{nmt81}rtetR-mph1(D1-302) alp4-myc:kanR mad2-GFP:his3*

KM10 *ade6-210 leu1-32 ura4-D18*

IY235 *lys1::P_{adh15}-rtetR-mCherry-$spc7_{1-666}$-9TE:ura4 tetO:kanR leu1::P_{nmt81}rtetR-mph1(D1-302) Mad3-GFP:his3*

IL266 *lys1::P_{adh15}-rtetR-mCherry-$spc7_{1-666}$-9TE:ura4 tetO:kanR $leu1^{+}$:P_{nmt81}rtetR-mph1(D1-302) Bub1-GFP:his3 bub3Δ::hygR*

KM1440 *lys1::P_{adh15}-rtetR-mCherry-$spc7_{1-666}$-9TE:ura4 $leu1^{+}$::tetO:kanR $leu1^{+}$:P_{nmt81}rtetR-mph1(D1-302) mad2-RFP::natR P_{adh21}-mad1-GFP::hygR*

IL322 *lys1::P_{adh15}-rtetR-mCherry-$spc7_{1-666}$-9TE:ura4 tetO:kanR $leu1^{+}$:P_{nmt81}rtetR-mph1(D1-302) GFP-atb2:leu+ mad2-RFP:natR*

KM1415 *lys1::P_{adh15}-rtetR-mCherry-$spc7_{1-666}$-9TE:ura4 tetO:kanR $leu1^{+}$::P_{nmt81}rtetR-mph1(D1-302) GFP-atb2:leu+ mad1-Δcc:hygR mad2-RFP:natR*

IL634 *lys1::P_{adh15}-rtetR-mCherry-$spc7_{1-666}$-9TE:ura4 tetO:kanR $leu1^{+}$:P_{nmt81}rtetR-mph1(D1-302) GFP-atb2:leu+ mad1-KAKA mad2-RFP:natR*

IL558 *lys1::P_{adh15}-rtetR-mCherry-$spc7_{1-666}$-9TE:ura4 tetO:kanR $leu1^{+}$:P_{nmt81}rtetR-mph1(D1-302) GFP-atb2:leu+ klp5Δ::ura4+ mad2-RFP:natR*

IL560 *lys1::P_{adh15}-rtetR-mCherry-$spc7_{1-666}$-9TE:ura4 tetO:kanR $leu1^{+}$:P_{nmt81}rtetR-mph1(D1-302) GFP-atb2:leu+ klp6Δ::ura4+ mad2-RFP:natR*

IL664 *lys1::P_{adh15}-rtetR-mCherry-$spc7_{1-666}$-9TE:ura4 tetO:kanR $leu1^{+}$:P_{nmt81}rtetR-mph1(D1-302) GFP-atb2:leu+ klp6Δ::ura4+ klp5Δ::ura4+ mad2-RFP:natR*

IL 540 *lys1::P_{adh15}-rtetR-mCherry-$spc7_{1-666}$-9TE:ura4 tetO:kanR $leu1^{+}$:P_{nmt81}rtetR-mph1(D1-302) GFP-atb2:leu+ klp2Δ::hygR mad2-RFP:natR*

IL 672 *lys1::P_{adh15}-rtetR-mCherry-$spc7_{1-666}$-9TE:ura4 tetO:kanR $leu1^{+}$:P_{nmt81}rtetR-mph1(D1-302) GFP-atb2:leu+ dhc1-D4::ura4+ mad2-RFP:natR*

SS19 *lys1::P_{adh15}-rtetR-mCherry-$spc7_{1-666}$-9TE:ura4 $leu1^{+}$:P_{nmt81}rtetR-mph1(D1-302) cen2::kanR:ura4+:lacO his7+::lacI-GFP P_{adh15}:RFP-atb2:natR*

Figure S4

IL245 *lys1::P_{adh15}-rtetR-mCherry-spc7$_{1\text{-}666}$-9TE:ura4 tetO:kanR leu1^{+}:P_{nmt81}rtetR-mph1(D1-302) bub1Δ::natR mad2-GFP:his3*

IL626 *lys1::P_{adh15}-rtetR-mCherry-spc7$_{1\text{-}666}$-9TE:ura4 tetO:kanR leu1^{+}:P_{nmt81}rtetR-mph1(D1-302) bub1CD1 mad2-GFP:his3*

IL386 *lys1::P_{adh15}-rtetR-mCherry-spc7$_{1\text{-}666}$-9TE:ura4 tetO:kanR mad2-GFP:his3*

KM10 *ade6-210 leu1-32 ura4-D18*

IY230 *lys1::P_{adh15}-rtetR-mCherry-spc7$_{1\text{-}666}$-9TE:ura4 tetO:kanR leu1^{+}:P_{nmt81}rtetR-mph1(D1-302) mad2-GFP:his3*

IY240 *lys1::P_{adh15}-rtetR-mCherry-spc7$_{1\text{-}666}$-9TE:ura4 tetO:kanR leu1^{+}:P_{nmt81}rtetR-mph1(D1-302) bub3Δ::hygR mad2-GFP:his3*

IL788 *lys1::P_{adh15}-rtetR-mCherry-spc7$_{1\text{-}666}$:ura4 tetO:kanR leu1^{+}:P_{nmt81}rtetR-mph1(D1-302) mph1Δ::natR mad2-GFP:his3*

IL719 *lys1::P_{adh15}-rtetR-mCherry-spc7$_{1\text{-}666}$:ura4 tetO:kanR leu1^{+}:P_{nmt81}rtetR-mph1(D1-302) mph1Δ::natR bub3Δ::hygR mad2-GFP:his3*

Supplemental references:

S1. Ding, D.Q., Yamamoto, A., Haraguchi, T., and Hiraoka, Y. (2004). Dynamics of homologous chromosome pairing during meiotic prophase in fission yeast. Dev Cell *6*, 329-341.

S2. Yamamoto, A., West, R.R., McIntosh, J.R., and Hiraoka, Y. (1999). A cytoplasmic dynein heavy chain is required for oscillatory nuclear movement of meiotic prophase and efficient meiotic recombination in fission yeast. J Cell Biol *145*, 1233-1249.

S3. West, R.R., Malmstrom, T., Troxell, C.L., and McIntosh, J.R. (2001). Two related kinesins, klp5+ and klp6+, foster microtubule disassembly and are required for meiosis in fission yeast. Mol Biol Cell *12*, 3919-3932.

S4. West, R.R., Malmstrom, T., and McIntosh, J.R. (2002). Kinesins klp5(+) and klp6(+) are required for normal chromosome movement in mitosis. J Cell Sci *115*, 931-940.

S5. Garcia, M.A., Koonrugsa, N., and Toda, T. (2002). Spindle-kinetochore attachment requires the combined action of Kin I-like Klp5/6 and Alp14/Dis1-MAPs in fission yeast. EMBO J *21*, 6015-6024.

S6. Heinrich, S., Sewart, K., Windecker, H., Langegger, M., Schmidt, N., Hustedt, N., and Hauf, S. (2014). Mad1 contribution to spindle assembly checkpoint signalling goes beyond presenting Mad2 at kinetochores. EMBO Rep *15*, 291-298.

S7. Vanoosthuyse, V., Valsdottir, R., Javerzat, J.P., and Hardwick, K.G. (2004). Kinetochore targeting of fission yeast Mad and Bub proteins is essential for spindle checkpoint function but not for all chromosome segregation roles of Bub1p. Mol Cell Biol *24*, 9786-9801.
